# Semantic change in adults is not primarily a generational phenomenon

**DOI:** 10.1073/pnas.2426815122

**Published:** 2025-07-28

**Authors:** Gaurav Kamath, Michelle Yang, Siva Reddy, Morgan Sonderegger, Dallas Card

**Affiliations:** ^a^Department of Linguistics, McGill University, Montreal, QC H3A 1A7, Canada; ^b^Mila—Quebec AI Institute, Montreal, QC H2S 3H1, Canada; ^c^Department of Psychology, McGill University, Montreal, QC H3A 1G1, Canada; ^d^Centre for Research on Brain, Language and Music, Montreal, QC H3G 2A8, Canada; ^e^School of Information, University of Michigan, Ann Arbor, MI 48109

**Keywords:** meaning change, sociolinguistics, variation, natural language processing, apparent time construct

## Abstract

A central question in the study of language change is whether or not such change is generational. If a language changes over time generation-by-generation, the process looks as follows: New generations of speakers introduce innovations, while older speakers conserve their usage patterns, and the language changes as new generations replace older ones. At the opposite extreme, language change could be a zeitgeist phenomenon, in which changes are universally adopted by speakers simultaneously, regardless of age or generational cohort. This paper asks this question in the context of word meaning change. We analyze meaning change in over 100 words across more than 7.9 million U.S. congressional speeches, to observe whether, when a word sense rises or falls in prominence, adult speakers from different generations uniformly adopt it, or those from older generations conserve their prior usage. Using language model-based word sense induction methods, we identify different senses of each word, and then model the prevalence of each of these word senses as a function of time and speaker age. We find that most words show a small but statistically significant effect of speaker age; across almost 140 y of Congress, older speakers typically take longer than younger speakers to follow changes in word usage, but nevertheless do so within a few years. Our findings indicate that despite minor age-based differences, word meaning change among mature speakers is likely not a generational process, but rather a zeitgeist process, in which older adult speakers can readily adopt new word usage patterns.

## Significance

This work presents a large-scale study of word meaning change that focuses not only on how meanings change over time but also how such changes are adopted by adult speakers of different ages. We analyze over 7.9 million U.S. Congressional speeches from 1873 to 2010, inducing distinct, interpretable word senses for over 100 words posited to have undergone meaning change during that period. We find a small but statistically significant effect of speaker age in determining semantic change; older speakers in the corpus are generally slower than younger counterparts to adopt the words’ new usage, but nevertheless do so quickly, even leading change in some cases. Our findings thus provide significant new insight into social processes of language change.

Languages change not only over a span of centuries but also within lifetimes. Montreal French, for example, saw its /r/ shift from apical to dorsal in the second half of the 20th century (see refs. 1 and 2), while ongoing changes in the use of the English “like” (see refs. 3 and 4) similarly illustrate such processes. These “within-lifespan” changes can be a key insight into fine-grained mechanics of language evolution over time. Yet despite this theoretical significance, many questions linger about the mechanics of within-lifespan change. Key among these is the role of speaker age: when language change is in progress, do older speakers preserve their own language usage patterns, or adapt to the change as it is happening?

If the answer to the question above is that older speakers do in fact preserve how they speak, then language change is generational (5). Under this lens, a language changes incrementally generation-by-generation. New generations introduce changes, and older generations, while continuing to remain inflexible to these changes, are gradually replaced; the language change becomes the norm as older generations fade away. In this case, older speakers can be thought of as almost like time-capsules, reflecting the language as it was used in the past. Conversely, if the answer to the same question is instead that older speakers adapt to ongoing language changes, then language change is a more uniform, generation-agnostic process, which Fruehwald (6) refers to as a zeitgeist effect. Under this lens, a language changes over time with little generational variation—when a language change is introduced, this change is adopted by speakers of all ages more or less uniformly, and the change becomes the norm once enough time has passed, regardless of which specific generations of speaker persist (6). Formally, generational change means the way someone speaks is a function of their year of birth, while zeitgeist change means it is a function of the (calendar) year—and while these are not the only possible models of change, they are the two most straightforward ones when it comes to linguistic change over time (see ref. 7 for discussion of other possible dynamics).

Deciding between such models of language change not only holds conceptual significance but also strong methodological implications on how we study the dynamics of language in society—the research focus of sociolinguistics. This is because prevalent methods in the field of sociolinguistics have involved using differences between speakers of different generations, recorded at the same point in time, to infer ongoing patterns of change over time. But this methodology, known as the apparent time construct (see refs. 5 and 7–9), relies on the assumption that adult speakers are consistent in their speech patterns through their lifetimes—in other words, that change is generational, and older generations preserve their original usage patterns.

Given the importance of the apparent time construct, subsequent work has aimed to shed light on whether speakers persist in their response to language change as they age (1, 2, 6, 9–14). This body of work has largely supported the notion that older speakers preserve their language use patterns, thereby suggesting that change is generational. However, most of the work that supports the theory relates to speech sounds (phonetic or phonological data), which may not accurately reflect processes of semantic change. Indeed, besides a small body of work that typically focuses on at most a handful of words (e.g., refs. 15–20), semantic change has largely been understudied in sociolinguistics, in part due to the difficulties in measuring it (16, 21).

As a result, despite small-scale studies of word meaning change vis-à-vis the apparent time construct (15, 19), there has been no large scale test of it in this context. Work on quotatives (e.g., the English “like”)—a semantic-pragmatic phenomenon—however, suggests speakers can change their language use well into adulthood (20, 22), while one analysis of progressives in 19th century English found multiple adult speakers adapt to an ongoing change (23). Similarly, although there is evidence from the language acquisition literature that people show a declining capacity to acquire phonological and morpho-syntactic aspects of language beyond childhood and late adolescence (see, e.g., refs. 24 and 25), other work has indicated an absence of such effects in vocabulary acquisition; adults continue learning new words throughout their life, with older speakers using richer vocabularies than younger counterparts (e.g., 26 and 27).

On the other hand, word meaning change has received significant attention in computational linguistics and natural language processing (NLP). As is typical of those communities, much of this work has been pursued in the form of specific tasks, such as the task of lexical semantic change detection e.g. refs. 28’^39^, see ref. 40 for a review. That is, given a set of words annotated in terms of how much they have changed over time, can these values be approximately predicted by some algorithmic method. Some NLP researchers have also theorized “laws” of semantic change based on large-scale analyses, such as the idea that common words change meaning more slowly (35), although these claims have been challenged by others (41). Crucially for our purposes, this body of work includes not only methods that detect semantic change over time but also those that induce interpretable word senses through which to understand this change (see refs. 31 and 36’^39^).

These conceptual and methodological motivations and opportunities prompt us to use language model-based word sense induction methods to investigate the role of speaker age in word meaning change. We adapt existing NLP methods for word sense induction and meaning change detection, and apply them to a historical corpus annotated for both time and speaker age. For the subset of senses that show clear change over our period of study, we then model word sense prevalence as a function of time and age. In doing so, we find evidence that older speakers are only slightly slower than younger ones to adapt to these changes, suggesting that word meaning change is primarily a zeitgeist process rather than a generational process. Our work thus contributes to two growing traditions within sociolinguistic research: computational sociolinguistics (e.g., refs. 42–45), and sociolinguistic approaches to semantics (e.g., refs. 46–49).

## Modeling Meaning Change

### Conceptual Approach.

We begin by conceptualizing word meaning change in terms of the use of different word senses. At a high level, this involves assuming that words may be used in systematically different ways, and that these manners of usage may be categorized into discrete “senses” of the word (see refs. 50–52 for more)—the word “bank,” for example, has multiple distinct meanings, one of which refers to a financial institution, and another which refers to the side of a river. Building loosely on prior work in a similar vein (15, 16, 31, 36), we take the relative prevalence of each of the senses of a given word to reflect the prominence of its various meaning(s) in popular usage; as a particular sense of a word enters into or departs from popular usage, we would expect to see mentions corresponding to that sense rising or falling as a proportion of all usages of the word, regardless of whether the popularity of the word itself is rising, falling, or staying the same. Although identifying a new sense of a word as it emerges is difficult, once a sense has been well established, we can look retrospectively to see when it first appeared (or disappeared). Consequently, to measure meaning change in a given word, we induce its distinct senses across an entire corpus, and then track how each of these senses account for more or less of the word’s use over time—whether through the appearance of a completely novel sense at some point in time, or simply a more gradual change in the relative frequencies of word sense usage.

### Data.

**Fig. 1.**
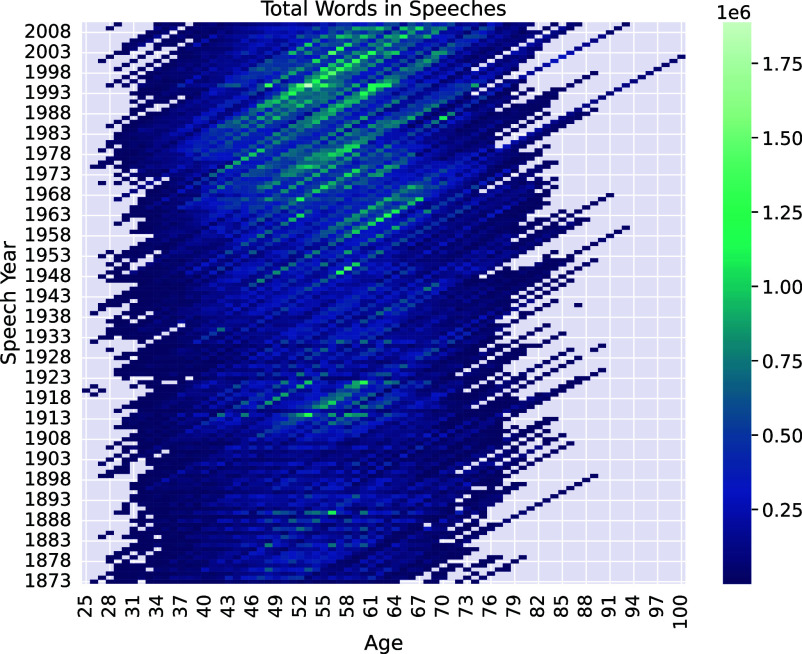
Heatmap of the U.S. Congressional Record corpus, showing total number of words by year of speech and speaker age, after filtering out procedural speeches. See *SI Appendix*, Figs. S1 and S2 for additional visualizations of the corpus.

A key barrier to studying meaning change in relation to age is the lack of large-scale corpora for which both year of publication and speaker or author ages are known. Prominent corpora (e.g., refs. 53–55) of historical texts, for example, lack either author bios or exact years of original publication. We therefore instead use historical records of political speeches: specifically, the Stanford Congressional Record dataset (56), which contains transcripts of all speeches made in both chambers (House and Senate) of the United States Congress between the 43rd and 111th Congresses (i.e., between 1873 and 2010). Much of this corpus comprises relatively formal speech from privileged members of society, many of whom share gender, ethnicity, and occupation. Nevertheless, the corpus offers analytic possibilities that are rare among linguistic corpora—it enables us to study text spoken in a consistent venue, across almost 140 y, by several thousand speakers, all of whose ages at the time of speech production are known. We enrich the dataset with speaker biodata taken from the @unitedstates project’s Congress Legislators dataset (57), and then use the classifier from Card et al. (58) to filter out speeches that are likely to be purely procedural (e.g., “without exception it is so ordered”). The final result is a corpus of over 7.9 million speeches totaling roughly 1.5 billion words, annotated for the date on which the speech was given, and the speaker’s age at the time. Fig. 1 shows the distribution of this corpus by speaker age and year of speech in terms of the total number of words spoken. As can be seen, the range of ages represented by this corpus spans ages 25 (Clarence McLeod, who was the youngest person elected to Congress at the time he was elected in 1920) to 100 (Strom Thurmond, who served in the Senate until just before his death at age 100 in 2003). See *SI Appendix* for more on the age- and time-distribution of our data, including the distribution of speaker ages for the target words discussed below.

### Word Sense Induction Method.

To focus on target words that are representative of word meaning change dynamics, we identify around 100 words deemed likely to have undergone meaning change over the 20th century. Twenty one of these are taken from existing work on meaning change detection (35, 59), while the remaining 85 were obtained by applying Hamilton et al.’s (35) methods to the Congressional Record dataset (see *Materials and Methods* for details).

Focusing on these target words, we use methods from Eyal et al. (38) to induce word senses from the data, and track their prevalence across the dataset. In brief, this method involves using a masked language model (see refs. 60 and 61) to predict likely replacements of a masked target word given the context in which it appears, and then using the distributions of these replacements to induce the different senses of the word. Unlike static word vectors, this approach allows us to infer the meaning of each specific use of a word in context, rather than just the average meaning across all usages. As Table 1 illustrates, this gives us clusters of replacements that, for the most part, intuitively reflect various senses of our target words. We then use the replacement data to estimate the probability of it being used in each of these senses, and filter out cases demonstrating little change in word sense probability over time, along with artifacts resulting from tokenization. Doing so leaves us with a total of 163 word senses from 81 unique words, with a median of 2 senses per word. We then model the likelihood of each of these word senses across the dataset, as described below.

**Table 1.** Examples of induced word senses, with selected in-context examples

Word	Top replacements in cluster	Intuitive sense	Selected in-context example
Alienation	Sale, possession, use	Legal (land)	*...existing law against the* alienation *of land by the Indians.* ^i^
Alienation	Fear, violence, isolation	Emotion	*...there is a general sense of* alienation *about our political process.* ^ii^
Articles	Items, things, goods	Object	*...for use in packing glassware and other easily breakable*articles.^iii^
Articles	Provisions, laws, acts	Legal	*...the*articles*for the government of the navy are hereby amended...* .^iv^
Articles	Papers, stories, reports	News	*...two* articles *by Sylvia Porter which dealt in a very perceptive way...* ^v^
Headed	Led, directed, chaired	Presided	*...harmful to working families, especially those* headed *by single parents.* ^vi^
Headed	Going, heading, moving	Going	*People are very worried about where we are* headed *economically.* ^vii^
Headed	Called, signed, titled	Entitled	*...the booklet published in 1968 is*headed “*problem growth*.”^viii^

^i^Augustus Bacon, February 7, 1907; ^ii^Robert Andrews, May 1, 1991; ^iii^Charles Vanik, September 10, 1979; ^iv^Edith Rogers, September 1, 1944; ^v^William Symington, March 21, 1968; ^vi^Betty McCollum, April 14, 2005; ^vii^Christopher Dodd, October 15, 2002; ^viii^James Allen, March 25, 1970.

### Modeling Usage Across Age and Time.

To model the adoption (or disadoption) of meanings as a function of both time and speaker age, while marginalizing over interspeaker differences, we fit generalized additive mixed models (GAMMs) (6, 63) with speaker-wise random effects to our word sense prevalence estimates, which accounts for the possibility of nonlinearities in trajectories of usage over time. For each word sense deemed to have undergone sufficient change in the corpus, we model the probability that, for a given year and speaker age, if the word were to be used, it would be used in that specific sense.

Given our a priori assumptions about the overall dynamics of language change—namely, that it is either a function of time or both time and age, but is unlikely to be a function of only age—our GAMMs model word sense probability as a function of time, with an interpretable offset for speaker age that we optimize. We use binomial GAMMs with a logistic link, which can be described by the following equation:^*^

$$p\left({\text{sense}}_{v}|m,y\right)=\sigma \left(f_{v}\left(y-\alpha_{v}\cdot \;\text{age}\left(m,y\right)\right)+\beta_{v}\left(m,y\right)\right),$$

where y is the year in which the speech was given, m is the member of Congress giving the speech, age(m,y) returns the age of speaker m in year y, $f_{v}\left(\cdot \right)$ is an arbitrary nonlinear smooth function modeling sense v, $\beta_{v}\left(m,y\right)$ is a random effect for each speaker and session combination to account for interspeaker and intersession differences, σ is a sigmoid function, returning a value between 0 and 1, and $p({\text{sense}}_{v}|m,y)$ is the probability that a particular sense is intended when the corresponding word is used by speaker m in year y. Finally, $\alpha_{v}$ is an optimized linear offset term for sense_*v*_, corresponding to an *age effect*, and the primary estimand of interest.

Intuitively, $\alpha_{v}$ corresponds to the degree to which age has an effect on word meaning change, which we model as constant across time and age. If $\alpha_{v}=0$, the word sense probability is captured purely as a function of the year of speech and individual speaker or session variation, and thus does not vary systematically with speaker age, representing a pure zeitgeist phenomenon. As $\alpha_{v}$ grows larger, age matters more, and older speakers are effectively offset in time, using words as if they were speaking in a different year. Larger values of $\alpha_{v}$ thus imply a slower acceptance of meaning change by older speakers. At $\alpha_{v}=1$, the effect of age completely offsets changes in time, implying that a speaker twenty years older than another would sound like a same-age speaker from twenty years earlier, plus any variation captured by the random effects $\beta_{v}\left(m,y\right)$, thus effectively modeling a generational effect. Note that we iteratively determine the optimal value of α for each sense by fitting a series of GAMMs. (See *Materials and Methods* for details.)

As validation of this approach, we also fit more unconstrained GAMMs on the data, which model word sense probability as any joint function of time, age, and interactions between the two.^†^ Doing so yields almost identical goodness-of-fit metrics, validating our more conceptually grounded and interpretable approach; see *SI Appendix* for the full set of model fit comparisons.^‡^

## Results

### Estimating Word Sense Prevalence Over Time.

**Fig. 2.**
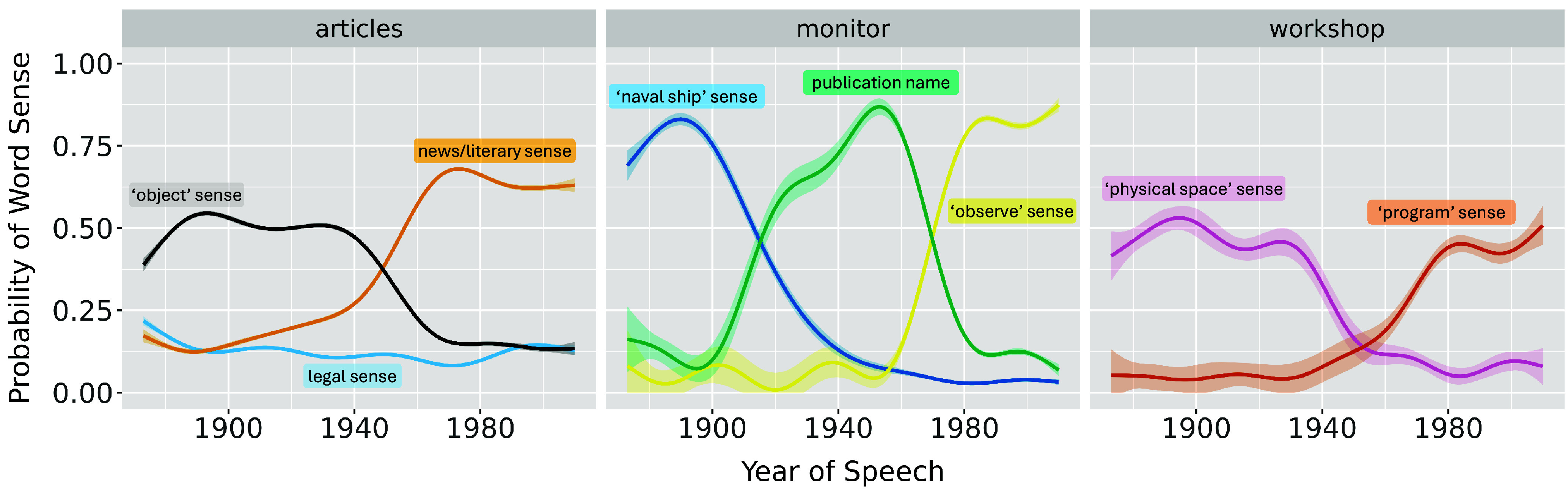
Examples of word sense probability over the time range of our corpus. Each panel shows the rise and fall of various senses of a single word, identified at the top of the graph. Each line is a generalized additive model (GAM) smooth calculated over our raw word sense probability data (62, 63), before taking into account any speaker information; uncertainty bands reflect varying amounts of data per word. Since word sense probabilities for any given word sum to 1, the rise of one word sense’s probability is accompanied by the fall of another’s. The probabilities in the examples above, however, do not sum to 1, as they only feature word senses corresponding to top two or three replacement clusters, ignoring the long tail of infrequent replacement clusters that generally accounts for the remaining probability space. See *Materials and Methods*, *Limitations*, and *SI Appendix* for more details.

Applying the previously described word sense induction methods to our data, we see evidence of several word senses rising and falling in usage prevalence. Fig. 2 shows a handful of examples—the literary sense of “articles,” for instance, steadily replaced the “objects” sense of the word, with a similar process taking place between the program-related and physical senses of “workshop.” In the case of the word “monitor,” we observe three distinct phases: first, when the word is mostly used to mean a type of ship; second, when the word is mostly used in the context of journal and newspaper names; and third, when the word is mostly used to mean “observe.” As noted in the previous section, most of the roughly 100 words we begin with show a significant amount of change in the use of at least one or two of their senses.

### Model Predictions by Age and Year.

**Fig. 3.**
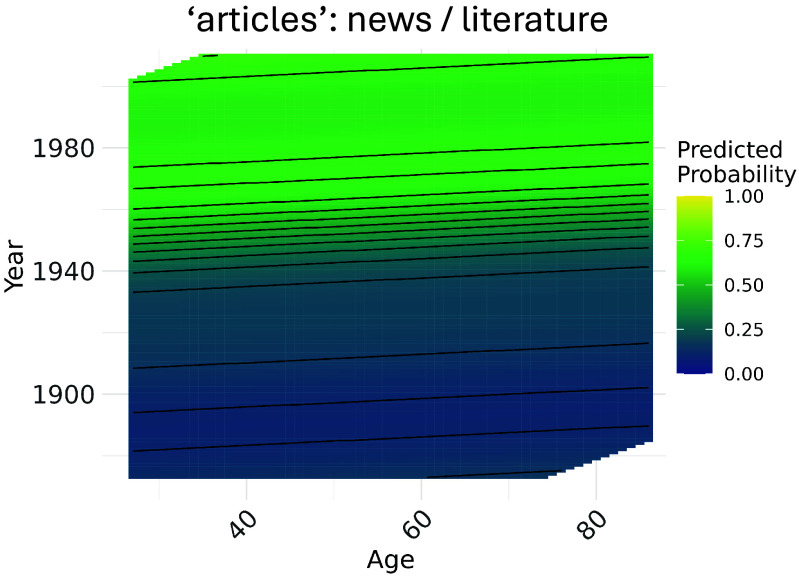
Heatmap of GAMM predictions across age and speech year, for the probability of “articles” in its literary or news-related sense. Lighter sections of the heatmap correspond to higher probabilities that, if the word is used, it is used in this sense; darker sections correspond to the opposite. Though the increasing use of “articles” in its contemporary sense is led by younger speakers in the decades around the 1940s, older speakers show only a minor lag in following this trend (missing sections of the grid correspond to age-year combinations that lacked sufficient input data).

Model predictions from the GAMMs we fit provide a picture of word sense likelihood across almost the entire range of years and ages in our dataset. Fig. 3 shows, as a heatmap, the full range of GAMM predictions across both time and age, for the probability of the word articles being used in its predominant contemporary sense. Crucially, the vast majority of variation present in Fig. 3 is across the dimension of speech year, albeit with some age-wise variation as well. The time-wise variation in probability corresponds to the gradual rise of the sense as visible in Fig. 2 (*Left* panel); assessing these predictions in the context of age as well, however, also reveals an apparent effect of the latter. This effect is directly captured by the model’s estimated α value, and reflected in the slope of model predictions—flatter slopes correspond to weaker age effects, and steeper slopes, the opposite.

**Fig. 4.**
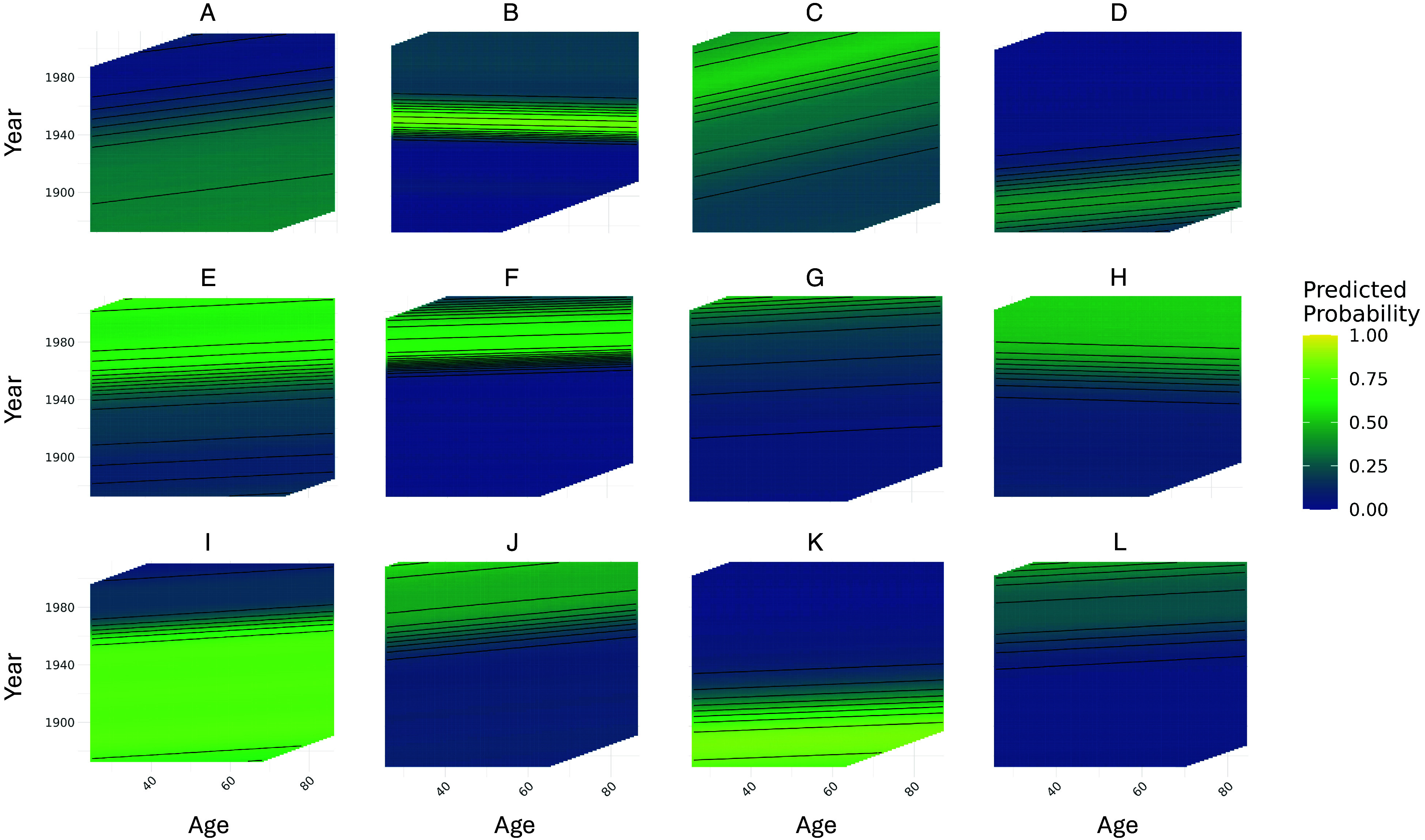
GAMM prediction heatmaps from a random sample of word senses (missing grid sections correspond to age-year combinations with insufficient data). (*A*): “tiny amount” sense of “particle”; (*B*): geopolitical sense of “satellite”; (*C*): “awful” as used in “awful lot”; (*D*): occupational sense of “folder”—person whose job consisted of folding material; (*E*): news- and literature-related sense of “articles”; (*F*): “purpose”/“point” sense of “thrust”; (*G*): financial sense of “forgiveness” (loan/debt); (*H*): sports-related sense of “coaches” (as opposed to vehicular); (*I*): political sense of “platforms”; (*J*): legislative sense of “package”; (*K*): “small warship” sense of “monitor”; (*L*): radio-related sense of “receiver.”.

Fig. 4 shows a random sample of such heatmaps from different word senses modeled. As it indicates, we find that, regardless of the nature of the change—whether it is the rise or fall of a word sense, and whether this change is gradual or rapid—the most common pattern visible is one of a small but typically positive age effect. Notably, in some cases, such as the use of “thrust” to mean “purpose,” or “satellite” in geopolitical contexts, we capture both the rise and the fall in prominence of a particular sense. (See *SI Appendix* for similar heatmaps for all word senses modeled.)

### Age Effects.

**Fig. 5.**
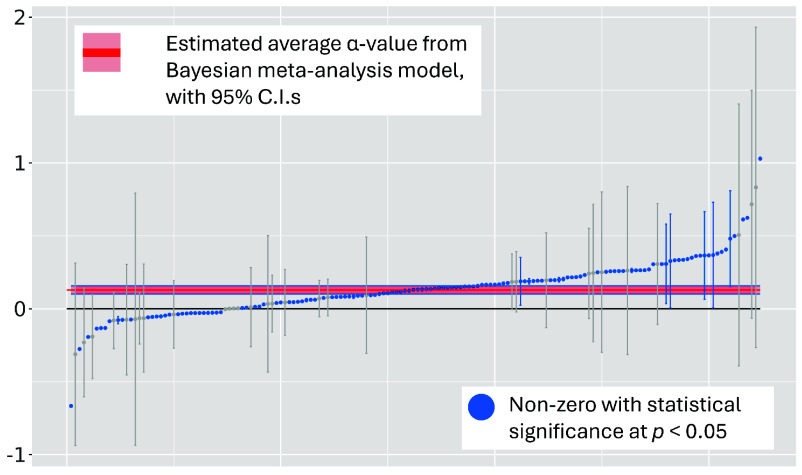
Plot of every α-estimate from our fitted GAMMs, each modeling a unique word sense, arranged in ascending order, with 95% CIs, and compared against the estimated average α-value from our Bayesian meta-analysis model. Color indicates whether estimates are statistically significant (p<0.05); wide CI all correspond to words with sparse data (see *SI Appendix* for more). Note the presence of a minority of negative α-values—these are cases where change appears to be led by older speakers, such as the geopolitical sense of “satellite” shown in panel *B* in Fig. 4.

Fig. 5 shows the full distribution of α-values from the GAMMs we fit, with 95% CI, and estimates highlighted to show statistical significance. To estimate the average age effect across our data, we fit a Bayesian meta-analysis model (65, Chapter 11.1) to the α-estimates and corresponding SE we obtain from our GAMMs. Doing so gives us a meta-estimate of α=0.13 (95% C.I: [0.10,0.16]) for the average age effect. As for individual word senses, most α-values are nonzero with statistical significance at p<0.05, and tend to fall either close to or slightly above zero.

On average, age therefore has a statistically significant but minor effect on word sense usage. As an illustration, an α-value of 0.15 suggests that a 60 y old in 1960 will match the same word sense likelihood of a 40 y old in 1957: in other words, an older speaker will lag a speaker who is 20 y younger by about three years. We find that 90% of statistically significant α-estimates lie within the range [−0.09,0.39]. At the higher extreme, our model predicts an older speaker will lag a speaker who is 20 y younger by about 8 y. At the lower extreme, older speakers in our corpus actually lead change very marginally, with speakers that are 20 y younger lagging by about 2 y. We find the latter dynamic in a minority of cases, such as the geopolitical use of “satellite”, shown as panel *B* in Fig. 4.

Although this degree of lag does show that younger speakers tend to employ new usage patterns before older speakers, the typically short time within which older speakers adapt to these changes—or in some cases, even lead it—is noteworthy, as it indicates that older speakers in our corpus are highly flexible to adopting new usage patterns within only a handful of years.

## Individual-Level Change

**Fig. 6.**
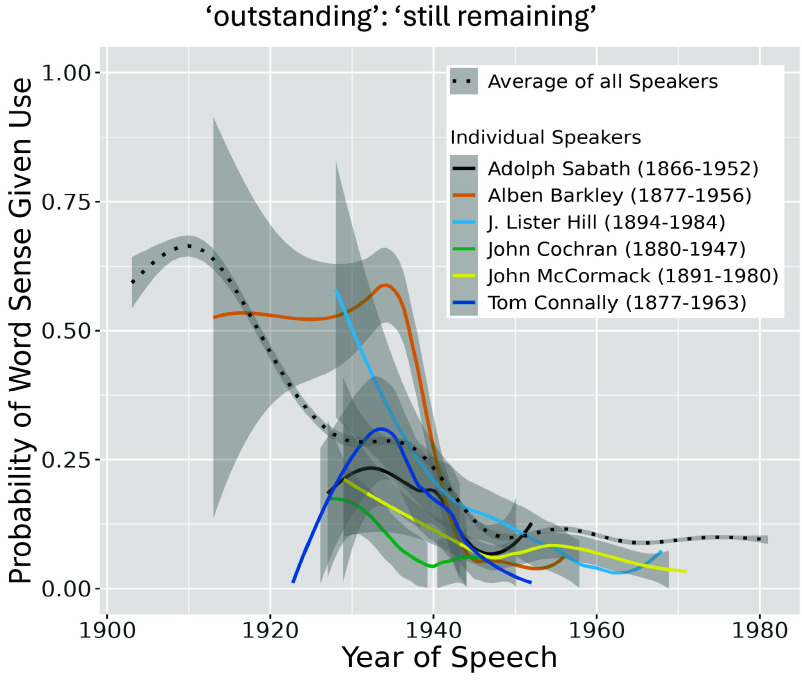
Probability of the word “outstanding” being used to mean still remaining/“continuing to exist,” showing how individual speakers change their usage patterns during their lifetime. Featured are the six speakers who used the word most frequently during the early and mid 20th century, a period in which the word saw significant usage change. As in Fig. 2, each line is a GAM smooth calculated over raw word sense probability data (62, 63), and uncertainty bands reflect varying amounts of data per word and sense. See *Materials and Methods*, *Limitations*, and *SI Appendix* for more details.

Assessing word sense usage change in our data at the level of individual speakers is difficult to conduct at scale, because it requires a speaker to have both given speeches across a sufficiently long time period, as well as used a specific word enough times across that duration. We therefore focus on a handful of prolific words and speakers across the dataset. We find that the same pattern observed at the population-level holds: speakers across different ages are quick to adopt new usage patterns and show significant variation within their lifetimes. Fig. 6 shows an example of this, for the “still remaining” sense of the word “outstanding.” Most of the prolific speakers featured show significant declines in their relative use of the word “outstanding” in this sense, and broadly follow the population-level trend. Though sometimes noisy, we find similar results for other words where enough data is available; these are reported in *SI Appendix*.^§^

## Discussion

Existing work asking whether speakers preserve their language use patterns over time has found that, for the most part, they often do, albeit with a minority of cases where speakers adopt an ongoing language change e.g., refs. 1, 2, 6, 9–13, and 66, see ref. 14 for a review. In turn, these studies support the idea that language change occurs between generations, and that the usage patterns of older speakers are representative of prior states of the language. Our results suggest that when it comes to word meaning change, this pattern does not hold, and that the phenomenon is more an instance of zeitgeist change than generational change. We find that although older speakers in our corpus are generally slower to employ novel word usage, they nevertheless adapt quite quickly—meaning that usage patterns change even within individual generations, and older speakers cannot be treated as representative of significantly earlier states of the language.

### Conceptual Implications.

In his seminal work on sound change, Labov (67) paints a rich picture of the mechanics of phonetic shifts—one in which language change from below involves successive generations of speakers in a subgroup responding to social pressures around their language use. Our findings indicate that word meaning change may often be a much faster process, with changes being adopted by speakers within only a few years. Consequently, our findings provide an alternate picture of language change to, as Fruehwald (6) writes, “a process of intergenerational incrementation with relatively stable intragenerational patterns.”

This contrast also echoes the possibility of a difference between language change dynamics for sound-based patterns on the one hand, and semantic or syntactic patterns on the other. Nevalainen et al. (68), for instance, build on work by Labov (69) to suggest that speakers may show more variability in their use of abstract structural patterns than phonological patterns, in part because the former are less likely to be socially evaluated. Similarly, Sankoff (7) offers a similar suggestion, though for different reasons—speculating that relatively “isolated” linguistic features, like individual words, may be easier for older speakers to learn than features that are implicated in more complex constraints and relationships within the speaker’s internal grammar, such as aspects of their sound system.

Our work finds further proof of such contrasts, but with a far greater degree of depth and scale—assessing semantic change in terms of not only a pair of morphemes or handful of words, but instead a set of over 160 word senses. Whether due to differences in how lexical and phonological features are socially evaluated, or differences in how they are embedded within a speaker’s internal grammars, we find that the dynamics of language change vis-à-vis word meaning change diverge from those of sound-based patterns. To a first approximation, word meaning change shows the opposite pattern to sound change: zeitgeist rather than generational change.

Finally, our findings raise important questions about language at the level of the individual. This is because our findings suggest that, even at an individual speaker level, older speakers are flexible in adopting new word meaning usage patterns. As a consequence, they bear some relevance to ideas of a sensitive or critical period for language acquisition (see, e.g., refs. 70–72).^¶^ While there is strong evidence that attaining native-like pronunciation of a second language is particularly difficult beyond a young age (e.g., refs. 24 and 73), and that similar constraints in acquiring morpho-syntax appear after late adolescence (e.g., refs. 25 and 74), our results complement studies on vocabulary acquisition, which reveal no such sensitive age period (26, 27, 75, 76). Though our findings concern the usage of words in new senses, rather than the introduction of new words to the lexicon, they offer a similar takeaway: speakers’ lexicons can remain flexible well beyond traditionally held critical periods of language acquisition.

### Methodological Implications.

The apparent time construct, traditionally a staple of sociolinguistics research, relies on the assumption that mature speakers are consistent in their language patterns (2, 5, 8, 9, 77). Crucially, the construct has allowed linguists to study change over time by examining language use in the present—getting around many of the challenges in sourcing historical linguistic data.

If older speakers adapt to ongoing language change, however, then apparent time differences between young and old speakers at a given point in time may fail to sufficiently reveal an ongoing change. This is in contrast to traditional concerns about the opposite—namely, that apparent time differences may be solely the result of age-grading effects, wherein differences between old and young speakers at one point in time are the result of young speakers linguistically innovating but older speakers reverting to conservative usage patterns, meaning no long term language change is truly in progress (5, 12). The little existing work on apparent time differences in word meaning (see refs. 15,18–19) has largely suggested that, where present, such differences are indeed indicative of ongoing change. Our findings do not run counter to this idea, but instead indicate that in the context of word meaning change, apparent time differences may actually underestimate the ongoing change, and may be only briefly observable during its course. A further implication of this is that it is likely not sufficient to study meaning change using linguistic data solely using data from the present. Instead, historical data remain necessary for such an inquiry, with this work showing one example of how to obtain such data.

### Limitations.

Due to the genre of our data, we only focus on change among adult speakers—minimum ages of membership in U.S. House of Representatives and Senate (which together form the U.S. Congress) are 25 and 30, respectively (78). It is adolescents, however, who are typically the drivers of linguistic change, being the first to innovate new language patterns (13, 79–81). As a result, although our results indicate that, on average, younger speakers exhibit change marginally sooner than older speakers, we are likely missing the original drivers of the word meaning change we see in our data. Furthermore, beyond the explicit linguistic limit of our data being confined to American English, political speech–which comes from a cohort of speakers that often covary in gender, ethnicity, and social class–does not fully represent the linguistic behavior of the wider language community it originates from. This observation is especially pronounced for the earlier years of our data, but also applies to its more recent periods, as the U.S. Congress has historically underrepresented women and ethnic minorities, and continues to do so despite increasing representation (82, 83).

On the side of technical implementation, our word sense induction methods also introduce a level of noise in detecting word senses. While they are robust at a high level, generating replacement clusters for most words that align with intuitive word senses (such as those shown in Table 1), they also generate a long tail of infrequent replacement clusters, and occasionally generate seemingly redundant clusters that both appear to point to the same word sense. Though this level of noise in our data does not affect our high-level findings, it does likely rule out more fine-grained analyses of individual word use instances. The full lists of replacement clusters are reported in *SI Appendix*, Table S1.

Finally, we would like to raise a more subtle point that invites further research. In this study, we measured the likelihood of different senses of a word being used, as an indicator of how the meaning of the word has changed. What exactly it means for a word to “change meaning,” however, may not be so straightforward. It is possible, for instance, that different senses of a word were always accessible, but that some start seeing more or less usage due to changing circumstances in the world and society at large—in other words, due to extralinguistic factors. This contrast is similar to what Hamilton et al. (84) identify as the difference between a “semantic drift” and “cultural shift.” Though beyond the scope of the current work, looking further into this distinction in the context of age- and time-dependent word meaning change marks a promising area for future research.

## Materials and Methods

### Data Processing.

We enrich the U.S. Congressional Record dataset from Gentzkow et al. (56) with speaker biodata from the @unitedstates project’s Congress Legislators dataset (57). By linking the two using speaker full names, states, and Congressional chamber, we were able to resolve a birth date for each speaker. We dropped data from any speakers that could not accordingly be mapped to unique biodata. To exclude purely procedural speeches (e.g., “Without exception it is so ordered”), we used the logistic regression classifier from Card et al. (58), which was trained for this purpose, and dropped any speeches with p≥0.5 of being procedural (see that paper for details).

### Term Selection.

Because most common words have relatively stable meanings over time, we began by selecting a set of terms that were likely to exhibit a high degree of meaning change over our time period of study. Some of those selected were discovered in previous computational work on meaning change (35), whereas others were taken from a competition dataset annotated for meaning change (59). To augment these, we identified additional terms from the Congressional Record itself, using the method of Hamilton et al. (35), which measures the distance between aligned pairs of static word vectors trained on separate corpora.

To find words that exhibited a large change over time within this corpus, we first divided the corpus into four time periods: 1890–1919, 1920–1949, 1950–1979, and 1980–2009. We then trained part of speech-tagged static word vectors on each time period using word2vec (85), and compared the first time period to the third, and the second to the fourth, using cosine similarity following a Procrustes alignment, as per Hamilton et al. (35). After inspecting the top 250 words from each comparison, and excluding those that appeared to be due to OCR errors or other aberrations, we kept lowercase nouns, verbs, and adjectives that appeared to be authentic examples of meaning change, giving us 81 additional candidate terms. For a complete list of terms used, please refer *SI Appendix*, *Initial List of Words with Sources*.

### Word Sense Induction.

We build on methods from Eyal et al. (38) to induce word senses across our data from masked language model predictions. For a target word w, we begin by identifying $S^{w}=s_{1},...,s_{m}$ speeches in the corpus that, after being tokenized using some masked language model ϕ’s tokenizer $T_{\phi }$, contain at least one use of $T_{\phi }\left(w\right)$, the tokenized target word. For each speech $s_{i}\in S^{w}$, we then identify $U_{i}^{w}=u_{i,1}^{w},...,u_{i,n}^{w}$ uses of $T_{\phi }\left(w\right)$ in $T_{\phi }\left(s_{i}\right)$, the tokenized speech. In turn, for each use $u_{i,j}^{w}$, we extract a context window of c tokens on either side of $T_{\phi }\left(w\right)$ in $T_{\phi }\left(s_{i}\right)$, replace $T_{\phi }\left(w\right)$ with a MASK token, provide this as input to the masked language model ϕ, and collect the estimated probabilities, according to the model, for all terms in the model vocabulary as possible replacements for the masked token. Doing so gives us a list of $R_{i,j}^{w}=r_{i,j,1}^{w},...,r_{i,j,k}^{w}$ most probable replacements for the target word in $u_{i,j}^{w}$, where k is a manually chosen hyperparameter, along with the logits associated with each replacement $r_{i,j,k}^{w}\in R_{i,j}^{w}$. Crucially, in this step, we discount both the target word itself and any word belonging to a predefined list of stopwords as a valid replacement, meaning that the k replacements *do not* include stopwords or the target word itself. However, we do not exclude morphological variants of the target word from the list of k most probable replacements.

Repeating this process for all speeches in $S^{w}$, we get $R^{w}=R_{1,1}^{w},...,R_{m,n}^{w}$ replacement lists. In our implementation, we use bert-large-cased (60) as our model ϕ, a c value of 50 tokens to either side, and a k value of 10 most probable replacements. The full list of stopwords used is given in *SI Appendix*.

We then use co-occurrence patterns of words within the replacement lists to cluster frequently co-occurring replacements with one another; these clusters in turn correspond to the intuitive meaning senses shown in Table 1. Following ref. 38, we begin by constructing an undirected graph $G_{w}$ for each target word. The nodes of $G_{w}$ are the set of unique replacement words generated in the previous step, $\left\{r^{w}:\exists R^{w}\;r^{w}\in R^{w}\right\}$. The edges of $G_{w}$ link any two replacements $r_{i,j,x}^{w}$ and $r_{i,j,y}^{w}$ that co-occur in some replacement list $R_{i,j}^{w}$, and are weighted by i) how frequently the two replacements co-occur, and ii) the logits assigned to the replacements by the model ϕ. The latter marks a departure from ref. 38, which only uses co-occurrence data; we found that including logit data as well eventually yielded more intuitive replacement clusters.

We then implement the Louvain community detection method (see refs. 38 and 86) to obtain our replacement clusters $C^{w}=C_{1}^{w},...,C_{z}^{w}$. As demonstrated in Table 1, the most prominent of these clusters in turn correspond to word senses. The value of z is not predetermined, and is a product of the community detection algorithm. For full details, please refer *SI Appendix*, *Word Sense Induction*.

### Tracking Word Sense Usage Over Time.

For each replacement cluster $C_{x}^{w}$ generated from a target word w, we track within each speech $s_{i}\in S^{w}$ the total proportion of replacements that belong to $C_{x}^{w}$, calculated as $|\left\{r^{w}:r^{w}\in R_{i,.}^{w}\wedge r^{w}\in C_{x}^{w}\right\}\left|\;/\;\right|\left\{r^{w}:r^{w}\in R_{i,.}^{w}\right\}|$. We take this to be an estimate of $p(C_{x}^{w}|w,s_{i})$, the probability that w was used in the sense corresponding to cluster $C_{x}^{w}$, given the speech $s_{i}$. To track how word sense usage has changed over time, we then compile this information by each year of speech, taking the mean $\bar{p(C_{x}^{w}|w,s^{\mathrm{year}}})$ of this probability across all speeches $S^{w|year}=s_{1}^{\mathrm{year}},...,s_{z}^{\mathrm{year}}$ made in that year which mention w. These mean year-wise probability values are what we use to filter out cases of inadequate change, and are what are plotted in Fig. 2. We take 20-y rolling averages of the mean year-wise probabilities, and filter out word senses that do not show a change of at least 0.3 across the whole range of years. Note that these rolling averages are only used to filter out word senses with minimal change; we fit all models on the raw, speech-level word sense probability data. We then also manually inspect each of the remaining replacement clusters, and identify “senses” that are purely artifacts of the masked language model’s tokenizer. Five such senses were found, for example, the word “dialog” being tokenized into “dial” and “og,” leading to a replacement cluster corresponding to uses of the word dialog. These senses were then excluded from further analysis.

### GAMMs.

We fit GAMMs on the speech-level cluster-wise replacement data, alongside the corresponding speech year and speaker age data from the speech. We use the mgcv package in R to fit binomial GAMMs with logistic link (87, 88). This approach allows us to incorporate both functions which flexibly and smoothly interpolate over continuous variables, such as year or age, as well as random effects, which are modeled as normally distributed with zero mean. Models were fit to optimize fREML, using the bam() function, with discretized covariates to lessen computation time (option discrete = TRUE).

For modeling explicit age effects, we use the model described in *Modeling Usage Across Age and Time*. Note that the random effect term described there, $\beta_{v}\left(m,y\right)$, is a sense-specific random effect term for a given speaker m in the session of Congress corresponding to year y. This allows for by-speaker and by-session variability, by allowing usage to differ between speaker–session pairs. It ignores higher-order dependencies: both speaker-specific variation (across sessions), and correlations in speaker behavior in successive sessions (autocorrelation), which proved computationally infeasible to include. See *SI Appendix*, *Generalized Additive Mixed Models* for further details.

To determine the optimal value of α for each sense, we iteratively fit a series of GAMMs—using the mle() function from the stats4 package to find the value of α resulting in the highest restricted maximum likelihood value from the GAMM, via the Brent optimization method (88, 89). In some cases, it was necessary to estimate $\alpha_{v}$ using a subset of the data, in order to avoid an excessive number of random effects, which made models computationally infeasible to optimize. In such cases, we only use data from the speakers and sessions in which a word is used most often, up to a maximum of 4,000 random effects. In either case, we take the α value for a given sense that results in the highest restricted maximum likelihood value, and then use that estimate to refit the GAMM to all the data. See *SI Appendix* for more on α-optimization, including the data loss during filtering, reported in *SI Appendix*, Table S2.

For comparison, we also fit less constrained GAMMs, which allow arbitrary age and year interactions, defined as follows:

$$p\left({\text{sense}}_{v}|m,y\right)=f_{v}\left(y\right)+g_{v}\left(\;\text{age}\left(m,y\right)\right)+h_{v}\left(y,\;\text{age}\left(m,y\right)\right)+\beta_{v}\left(m,y\right).$$

The functions $f_{v}\left(\cdot \right)$ and $g_{v}\left(\cdot \right)$ are arbitrary nonlinear functions which are marginal smooths over *year* and *age*, respectively; $h_{v}\left(\cdot ,\cdot \right)$ is an arbitrary function which is a tensor product over *year* and *age*; m, y, age(·) and $\beta_{v}$ represent member of congress, year, age, and sense-specific random effects as defined before. Note that this unconstrained GAMM essentially allows an arbitrary joint function of year and age, parameterized (as a tensor product interaction using ti() from mgcv) so that “average” effects of year and age can be detected.

To compare how well the constrained and unconstrained models fit the data, we look at R-squared values, fREML values, and normalized $L^{2}$ distances between the resulting fits over years and ages. Adjusted R-squared values from our GAMMs provide evidence that these models fit the data well—both sets of models produce a median adjusted R-squared value of 0.57. Normalized $L^{2}$ distances show greater variation, and are given in *SI Appendix*, Table S1, along with side-by-side comparisons for a large random sample of word senses. Qualitatively, most unconstrained GAMMs show some degree of nonlinearity but otherwise are closely approximated by the constrained models with an explicit age effect.

### Bayesian Meta-Analysis Model.

We implement our Bayesian meta-analysis model in R using the brms front-end (90) to Stan (91). The model (see 65, Chapter 13.1) can be defined as follows:

$$\begin{array}{c} \begin{array}{cc} \alpha_{v} & ∼\;\text{Normal}\left(\zeta_{v},\epsilon_{v}\right)\;n=1,...,N_{\text{sense}}\\ \zeta_{v} & ∼\;\text{Normal}(\zeta ,\tau )\\ \zeta & ∼\;\text{Normal}(0,1)\\ \tau & ∼\;{\text{Normal}}_{+}\left(0,1\right), \end{array} \end{array}$$

where $\alpha_{v}$ is the observed age effect estimate for a given word sense; $\epsilon_{v}$ is the SE for this estimate; $\zeta_{v}$ is the “true” age effect value for that specific word sense; ζ is the overall true age effect value (i.e., an overall effect that each word sense-specific true α-estimate is derived from); and τ is the SD of true α-estimates between word senses. The distributions for ζ and τ (last two lines) are weakly informative regularizing priors. We use this model to estimate and report ζ, the true, average age effect. See *SI Appendix*, Fig. S10 for the full code used to fit the model.

## Supplementary Material

Appendix 01 (PDF)

## Data Availability

Data and code from this study are available on GitHub (https://github.com/McGill-NLP/meaning-change) (92).

## Footnotes

^*^These data would in theory be best modeled using a multinomial model for each word, with multiple age effect terms to account for different sense-wise age effects. We use sense-wise binomial GAMMs instead, as jointly optimizing multiple age effect terms ($\alpha_{v}$) within a single multinomial GAMM was computationally infeasible at the scale of our data. See *SI Appendix* for further discussion.

^†^Note that this is largely similar to Fruehwald’s (6) method of fitting GAMMs on age- and time-annotated speech data to model phonological change. Unlike us, however, Fruehwald fits models with speaker years of birth instead of age, and uses somewhat different exact model specifications.

^‡^The reader may wonder why we choose not to simply fit these unconstrained GAMMs in the first place, and only report their results. The reason has to do with interpretability—the latter provide measures of goodness of fit and statistical significance, but not straightforwardly interpretable effect size measures. On the other hand, our approach, which includes an explicit age effect coefficient as a linear offset, provides us a directly interpretable measure of age effects in meaning change, although it involves the simplifying assumption that $\alpha_{v}$ is constant over time and age. Indeed, our approach was partly inspired by exploratory analysis of unconstrained GAMMs, which seemed to suggest that this more interpretable method could approximate the same fit of the data as the unconstrained GAMMs. See ref. 64 for more on interpreting GAMMs.

^§^We also include in *SI Appendix* similar plots for these words, but with generational cohorts instead of individual speakers. These confirm the pattern implicit in the age-time heatmaps in Figs. 3 and 4: generational cohorts (split by decade of birth) show significant variation in word sense usage over time.

^¶^The term “critical period” can be conceptually loaded, and sometimes refer to ideas of neural plasticity and hard cutoffs in second language acquisition capabilities beyond a young age—see Birdsong (72) for examples of this debate. Here, we make no such claims, and use the term simply to refer to the fact that younger language learners typically attain native-like proficiency more easily than older counterparts.
