# Supplementary material for: Semantic change in adults is not primarily a generational phenomenon
Source: Proc Natl Acad Sci U S A. 2025 Jul 28;122(31):e2426815122. doi: 10.1073/pnas.2426815122 (PMC12337318; doi:10.1073/pnas.2426815122)
Supplement: Supplementary file 1 — Appendix 01 (PDF) [file pnas.2426815122.sapp.pdf]

Supporting Information for

Semantic Change in Adults is Not Primarily a Generational Phenomenon

Gaurav Kamath, Michelle Yang, Siva Reddy, Morgan Sonderegger and Dallas Card

Gaurav Kamath.
E-mail: gaurav.kamath@mail.mcgill.ca

This PDF file includes:

Supporting text
Figs. S1 to S61
Tables S1 to S2
SI References

Supporting Information Text

Corpus Size and Distribution

Figures S1 and S2 provide additional details of the size and distribution of our corpus after filtering out procedural speeches. Figure S1 shows the distribution of total word counts by speaker age, for each decade in the corpus. Figure S2 shows, for a sample of 30 of our target words, the distribution of word mentions by speaker age.

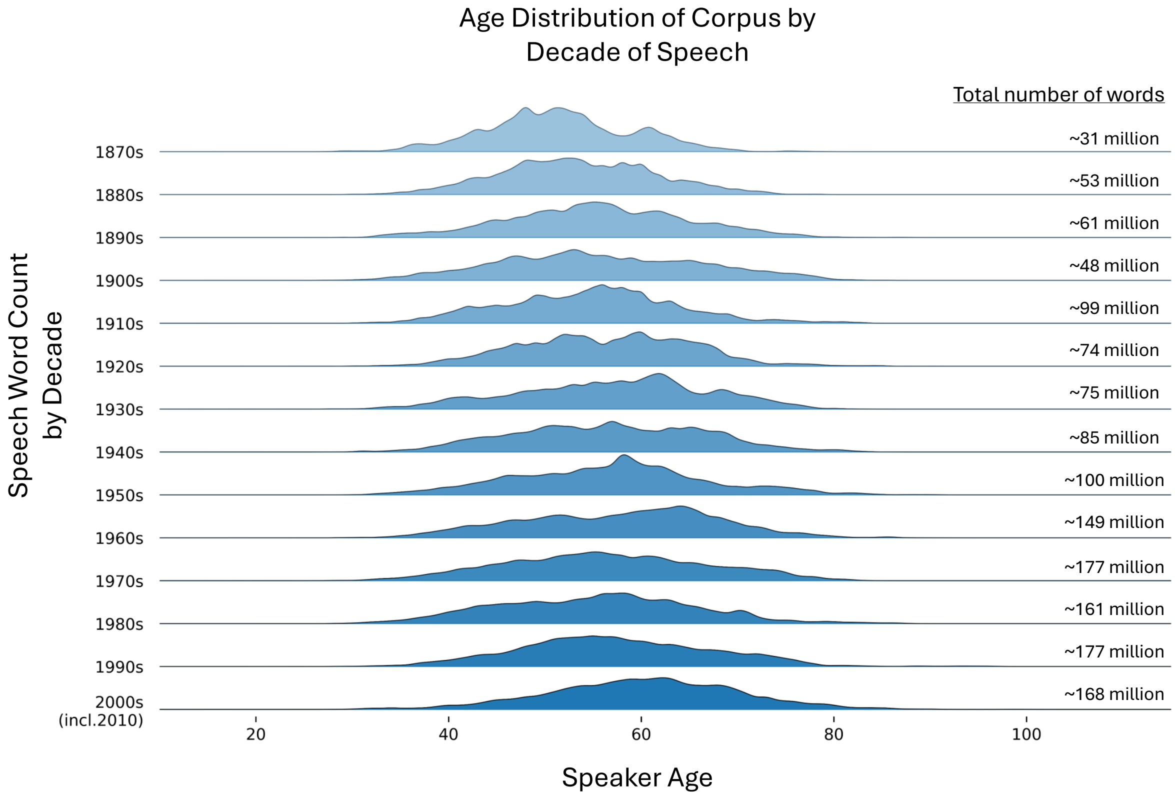

Fig. S1. Corpus size (measured in terms of total word count) by speaker age across each decade in the corpus. Note that there has been a gradual rise in the amount of speech delivered in the US Congress over the past century and a half. While the median speaker age is typically between 50 and 60, the scale of the data means that a much wider age range is nonetheless represented with several million words.

Initial List of Words with Sources

Below is the full list of 106 words we select as having likely undergone meaning change over the period our corpus covers, along with the source from which we collect or identify the word.

Taken from SemEval 2020 Task 1 (1): plane, tip, prop, graft

Taken from Hamilton et al. (2): gay, fatal, awful, nice, broadcast, monitor, record, guy, call, started, headed, calls, actually, wanting, check, starting, major

Identified by applying methods from Hamilton et al. (2) on our corpus: outstanding, astronomical, ceiling, shift, high, thrust, challenges, massive, fuel, articles, dial, recession, arraignment, receiver, maneuvering, impact, target, segregation, lunatic, receiving, rendezvous, pregnant, leads, implicit, retarded, receivers, locator, alienation, demonstrators, usage, appraising, security, cutoff, subversive, forgiveness, folders, workshops, drives, summit, background, stewards, organ, tending, needle, playing, coaches, particle, sterling, fixation, cap, masters, indexed, powerhouse, forensic, satellite, clearance, innovation, pleased, validation, match, hybrid, package, concerns, fabulous, sector, shark, caps, glue, threshold, amorphous, substance, signal, promiscuous, extremity, fantastic, pets, robust, projection, wired, workshop, complex, shorthand, platforms, fans, fix

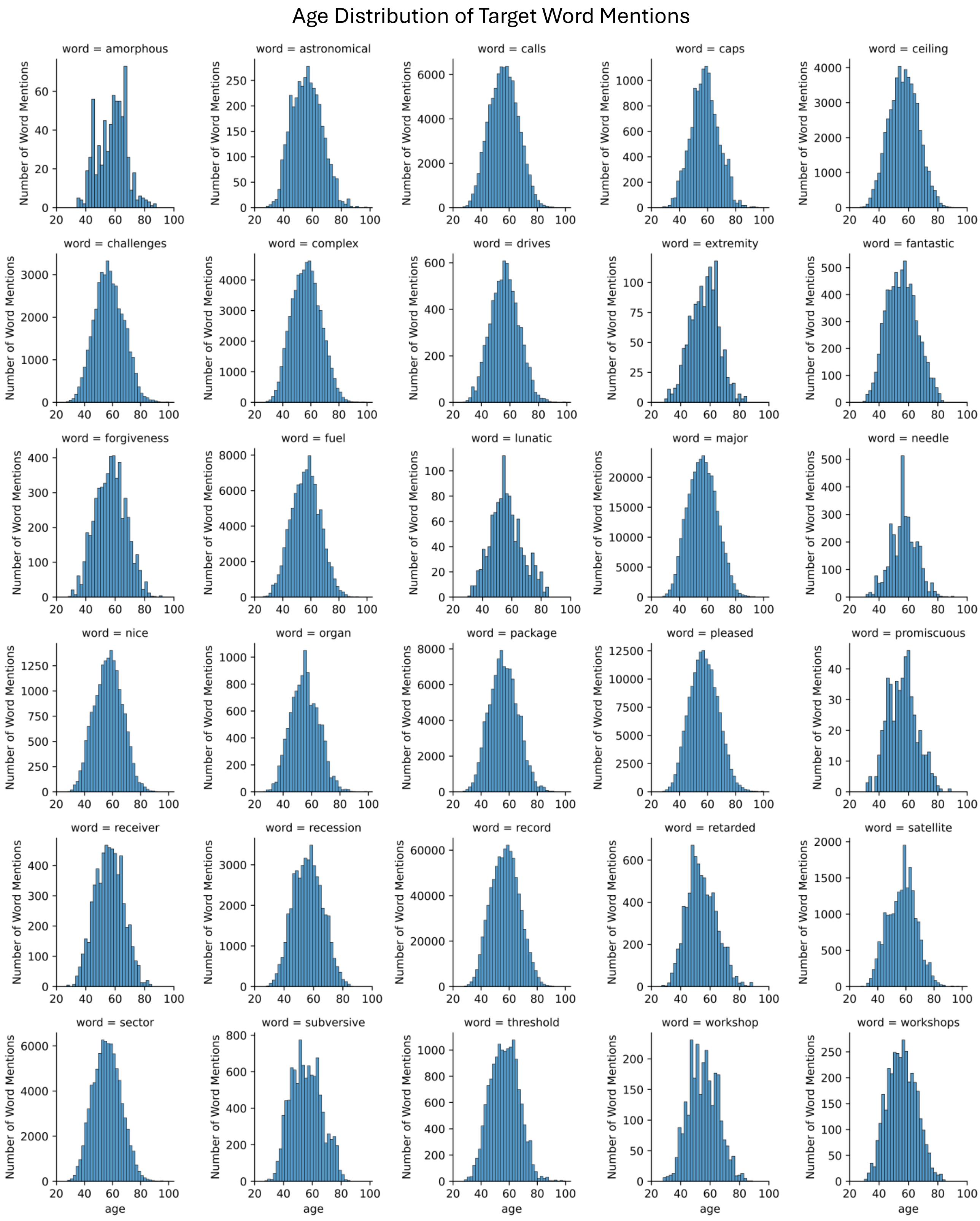

Fig. S2. Histograms showing, for a random sample of our target words, the distribution of word mentions by speaker age. While some words (like 'amorphous') see relatively few mentions across the corpus, others (like 'major' or 'record') see hundreds of thousands of mentions. As with the overall word count distribution of the corpus (see Figure S1), these distributions tend to be centered around middle-aged speakers, but the scale of the data means that a broader age range of speakers is still represented by a large number of mentions for most words.

Target Word Substitution Generation

List of Stopwords. The full list of stopwords used in the substitution generation process is provided below:

i, me, my, myself, we, us, our, ours, ourselves, you, your, yours, yourself, yourselves, he, him, his, himself, she, her, hers, herself, it, its, itself, they, them, their, theirs, themselves, what, which, who, whom, this, that, these, those, am, is, are, was, were, be, been, being, have, has, had, having, do, does, did, doing, will, would, shall, should, can, could, may, must, might, ought, isn, aren, wasn, weren, hasn, haven, hadn, doesn, don, didn, won, wouldn, shan, shouldn, cannot, couldn, mustn, let, a, an, the, and, but, if, or, because, as, until, while, of, at, by, for, with, about, against, between, into, through, during, before, after, above, below, to, from, up, down, in, out, on, off, over, under, again, further, then, once, here, there, when, where, why, how, all, any, both, each, few, more, most, other, some, such, no, nor, not, only, own, same, so, than, too, very

Word Sense Induction

Louvain Clustering Implementation. We use the `python-louvain` Python package to implement Louvain clustering on our word replacement data. The clustering is non-deterministic, and exact replacement clusters can vary slightly with different random seeds (though, qualitatively, we found clusters across random seeds would still largely align with the same intuitive word senses). For all clustering, we therefore use a common random seed, `3535`.

Word Replacement Clusters. In the main paper (see e.g. Table 1 and Figure 2 in the main paper), we report word replacement clusters in terms of the intuitive word senses they correspond to. The raw output of our clustering algorithm, however, is simply a numbered list of clusters, each containing a unique list of replacement words. For example, the cluster which we report as intuitively corresponding to the 'going' sense of 'headed' is Cluster 7 among the replacement clusters of the word.

On average, roughly 15 such clusters are generated from each word's replacements (mean=15.49, median=15); as we mention in the paper, however, the vast majority of these are extremely low frequency, and are likely noise. These low frequency clusters are filtered out when we filter for word senses displaying high enough usage and change, but can be found in the data and code repository for this paper, at github.com/grvkamath/meaning-change. Below is the full list of word replacement clusters after this filtering step, with the most frequent replacement words in each cluster—indicative of the intuitive word sense that the replacement cluster corresponds to.

Table S1. Words and senses included in our analysis, along with estimated α values with 95% C.I.s (estimate ± 1.96 SE), and the normalized L^2 distance between predictions from our explicit age effect GAMMs and our unconstrained GAMMs (see Goodness of Fit Measures). Cluster numbers match those shown in plots below.

Word	Cluster Number	Top Replacements	α-Estimate	95% C.I.	L^2-Distance
alienation	1	sale possession use acquisition	0.07	[-0.06, 0.19]	0.41
alienation	8	fear violence anger death	0.31	[0.04, 0.58]	0.05
amorphous	4	complex entire dangerous complicated	0.83	[-0.27, 1.93]	0.29
amorphous	2	big good important great	-0.07	[-0.94, 0.79]	0.46
amorphous	3	pure solid liquid synthetic	0.08	[-0.05, 0.20]	0.83
appraising	1	assessing determining considering evaluating	0.03	[-0.43, 0.50]	0.07
appraising	5	customs police certain first	0.09	[-0.31, 0.49]	0.26
arraignment	0	examination opinion criticism charge	0.33	[0.00, 0.65]	0.37
arraignment	12	trial arrest execution detention	0.37	[0.06, 0.67]	0.11
articles	2	papers stories reports letters	0.14	[0.14, 0.14]	0.04
articles	3	items things goods products	0.08	[0.08, 0.08]	0.07
astronomical	1	scientific actual various space	-0.28	[-0.28, -0.27]	0.03
astronomical	4	enormous huge massive unprecedented	-0.08	[-0.10, -0.05]	0.01
awful	0	enormous entire amazing incredible	0.61	[0.61, 0.62]	0.12
background	4	body past world house	0.26	[0.25, 0.27]	0.05
broadcast	0	television radio broadcasting tv	0.15	[0.14, 0.15]	0.07
broadcast	5	everywhere back widely forth	0.19	[0.17, 0.21]	0.03
broadcast	12	show program broadcasts report	0.12	[0.11, 0.12]	0.13
cap	5	limit ceiling bill tax	0.15	[0.15, 0.15]	0.12
caps	1	limits restrictions rules controls	0.05	[0.05, 0.05]	0.04
caps	0	bars heads mills hats	0.33	[0.33, 0.34]	0.25
ceiling	1	limit level ceilings budget	0.01	[0.01, 0.02]	0.03
ceiling	11	floor wall dome line	0.09	[0.09, 0.09]	0.04
ceiling	2	maximum price minimum total	-0.04	[-0.04, -0.04]	1.03
ceiling	3	limitation bill requirement issue	0.09	[0.09, 0.10]	0.04
challenges	3	needs things demands requirements	0.08	[0.08, 0.09]	0.06
challenges	0	questions concerns targets limits	-0.03	[-0.03, -0.02]	0.05
challenges	5	problems issues difficulties risks	0.23	[0.23, 0.23]	0.01

clearance	1	warrant briefing assurance alert	-0.02	[-0.03, -0.02]	0.03
clearance	3	housing removal development construction	-0.03	[-0.04, -0.03]	0.09
coaches	0	fans athletes men teachers	-0.07	[-0.08, -0.07]	0.02
coaches	1	cars carriages owners wagons	0.04	[0.03, 0.05]	0.04
concerns	1	worries concerned involves includes	0.12	[0.12, 0.13]	0.24
concerns	0	interests things people companies	0.16	[0.16, 0.16]	0.05
concerns	2	questions concern fears views	0.07	[0.07, 0.08]	0.02
cutoff	4	starting end start due	0.26	[0.25, 0.26]	0.13
cutoff	1	line point deal river	0.17	[0.16, 0.17]	0.10
cutoff	3	reduction loss withdrawal program	0.14	[0.14, 0.15]	0.03
demonstrators	7	students police persons officers	0.19	[-0.02, 0.39]	0.53
dial	11	mo hand blog end	-0.67	[-0.68, -0.66]	0.36
drives	4	drive efforts programs campaigns	0.01	[0.00, 0.01]	0.07
drives	1	makes takes brings runs	-0.06	[-0.06, -0.05]	0.06
extremity	8	arm leg body one	0.04	[-0.18, 0.27]	0.13
fabulous	0	great good wonderful real	0.50	[0.49, 0.51]	0.02
fans	3	fan ones lights cars	0.26	[0.25, 0.28]	0.08
fantastic	0	great good wonderful incredible	0.25	[0.25, 0.26]	0.04
fantastic	1	ridiculous absurd impossible true	0.19	[0.19, 0.19]	0.09
fixation	0	production use reduction extraction	0.48	[0.15, 0.81]	0.11
folders	6	books papers documents pages	0.51	[-0.39, 1.40]	0.05
folders	2	men persons officers members	0.25	[-0.30, 0.80]	0.26
forensic	2	great personal intellectual mental	0.37	[0.01, 0.73]	0.07
forgiveness	5	relief assistance payments credit	0.13	[0.13, 0.14]	0.02
forgiveness	0	forgive pardon redemption mercy	0.14	[0.14, 0.15]	0.13
glue	1	paper oil plastic water	0.20	[-0.13, 0.52]	0.11
glue	7	force strength power anchor	-0.19	[-0.48, 0.10]	0.03
graft	0	profit work put force	0.31	[0.30, 0.31]	0.12
graft	8	corruption fraud crime bribery	0.15	[0.14, 0.15]	0.10
guy	3	man person boy kid	0.31	[0.30, 0.31]	0.01
guy	8	john james george senator	0.36	[0.36, 0.37]	0.14
headed	0	led directed chaired represented	0.41	[0.40, 0.41]	0.10
headed	7	going heading looking moving	0.39	[0.39, 0.39]	0.04
hybrid	13	electric gasoline gas hydrogen	0.04	[0.04, 0.05]	0.09
implicit	2	full great complete total	0.17	[0.17, 0.18]	0.18
implicit	0	express right verbal faithful	0.11	[0.11, 0.11]	0.19
implicit	6	contained included inherent found	0.13	[0.13, 0.14]	0.07
indexed	3	used made done changed	-0.03	[-0.03, -0.02]	0.26
indexed	0	adjusted set designed ready	0.22	[0.21, 0.22]	0.05
innovation	3	growth progress power life	0.08	[0.08, 0.09]	0.01
innovation	0	change improvement invention action	0.17	[0.16, 0.17]	0.01
locator	10	information support care search	-0.06	[-0.24, 0.11]	0.01
locator	5	radio warning radar emergency	-0.08	[-0.27, 0.11]	1.43
locator	2	owner person man applicant	0.18	[-0.01, 0.38]	0.04
lunatic	2	insane mental political general	-0.07	[-0.45, 0.30]	1.01
major	2	great significant key huge	-0.06	[-0.06, -0.06]	0.01
major	3	general principal total captain	0.06	[0.06, 0.07]	0.01
maneuvering	4	training battle military new	0.10	[0.09, 0.11]	0.15
maneuvering	1	action power debate pressure	0.15	[0.15, 0.16]	0.08
masters	2	graduate doctoral doctorate college	0.15	[0.15, 0.16]	0.02
match	6	make meet use get	-0.03	[-0.03, -0.03]	0.01
match	9	limit bound peer age	0.16	[0.16, 0.17]	0.36
monitor	2	study observe watch examine	0.03	[0.03, 0.03]	0.01
monitor	0	monitors ship union line	0.11	[0.10, 0.11]	0.02
monitor	1	review report journal reporter	-0.03	[-0.03, -0.03]	0.04
needle	11	blood drug foreign cultural	-0.04	[-0.04, -0.04]	0.26
organ	0	body newspaper journal publication	-0.13	[-0.14, -0.13]	0.81
organ	5	organs blood heart kidney	0.19	[0.19, 0.20]	0.01
outstanding	1	excellent important extraordinary exceptional	-0.03	[-0.03, -0.03]	0.01
outstanding	2	great good remarkable distinguished	-0.19	[-0.20, -0.19]	0.05
outstanding	7	current made existing issued	-0.14	[-0.14, -0.13]	0.04

package	1	thing bundle one box	0.22	[0.22, 0.22]	0.01
package	0	program plan act budget	0.26	[0.26, 0.26]	0.02
particle	4	bit thing second day	0.36	[0.36, 0.37]	0.14
particle	8	nuclear ion electron energy	0.13	[0.12, 0.15]	0.15
particle	2	lot degree moment word	0.38	[0.37, 0.39]	0.06
pets	9	animals cats things livestock	-0.04	[-0.27, 0.19]	0.22
pets	4	members men friends property	-0.06	[-0.44, 0.31]	0.07
plane	3	level ground line basis	0.19	[0.19, 0.20]	0.01
plane	2	aircraft jet fighter bomber	0.22	[0.21, 0.22]	0.02
platforms	1	systems ships things vessels	0.09	[0.09, 0.10]	0.02
platforms	0	papers parties conventions campaigns	0.16	[0.16, 0.17]	0.04
powerhouse	7	great new main player	-0.23	[-0.60, 0.14]	0.72
powerhouse	0	pipeline powerplant weir aqueduct	0.04	[-0.16, 0.23]	0.92
powerhouse	1	dam reservoir plant station	0.24	[-0.07, 0.55]	0.16
pregnant	11	single poor little new	1.03	[1.02, 1.04]	0.01
pregnant	2	young black elderly many	0.34	[0.33, 0.34]	0.05
pregnant	4	born living filled now	0.26	[0.26, 0.27]	0.16
projection	1	development review use extension	0.26	[0.25, 0.26]	0.24
promiscuous	1	public general illegal direct	0.25	[-0.23, 0.72]	0.27
receiver	7	receivers one carrier station	0.15	[0.14, 0.16]	0.08
receivers	15	directors trustees wards survivors	0.37	[0.36, 0.37]	0.08
receivers	8	stations sets equipment systems	0.25	[0.24, 0.26]	0.08
recession	0	year problem period thing	-0.00	[-0.01, 0.00]	0.07
rendezvous	1	place station home base	0.31	[-0.11, 0.72]	0.07
rendezvous	14	meeting relationship connection problem	0.19	[0.02, 0.35]	0.47
retarded	4	affected delayed stopped made	0.08	[0.08, 0.09]	0.01
retarded	11	disabled ill handicapped impaired	0.12	[0.12, 0.13]	0.12
satellite	3	satellites space system mission	-0.05	[-0.05, -0.04]	0.91
satellite	4	cable television radio communications	-0.09	[-0.09, -0.08]	0.90
satellite	5	new small national foreign	-0.05	[-0.06, -0.05]	0.88
satellite	0	member world government state	-0.13	[-0.14, -0.13]	0.06
sector	4	world companies industries community	-0.08	[-0.08, -0.07]	0.65
sector	6	economy economics directly ever	0.00	[-0.00, 0.01]	0.91
sector	0	part segment area side	-0.03	[-0.03, -0.02]	0.13
segregation	1	slavery education color blacks	0.06	[0.06, 0.07]	0.22
segregation	0	division separation exclusion equality	0.00	[-0.00, 0.01]	0.17
segregation	2	discrimination violence racism poverty	0.26	[0.26, 0.27]	0.07
shark	13	broker man agent manager	0.26	[-0.31, 0.84]	0.02
shorthand	7	goddamn except swamp burden	0.01	[-0.26, 0.28]	0.71
shorthand	0	general newspaper news court	0.72	[-0.07, 1.50]	0.28
signal	0	great public special first	0.26	[0.26, 0.27]	0.18
signal	3	message warning note letter	0.01	[0.01, 0.02]	0.06
signal	2	signals bell light flag	-0.03	[-0.03, -0.02]	0.29
sterling	5	john william thomas james	0.13	[0.13, 0.14]	0.85
sterling	1	great good strong high	0.35	[0.35, 0.35]	1.27
stewards	0	members men managers employees	-0.03	[-0.04, -0.02]	0.44
stewards	14	staff beds wards ships	0.20	[0.19, 0.22]	0.04
stewards	3	management police fire engineers	0.09	[0.08, 0.10]	0.69
stewards	5	citizens owners guardians users	0.09	[0.07, 0.10]	0.00
substance	0	effect part fact general	0.27	[0.27, 0.27]	0.07
subversive	0	destructive part one outside	0.05	[0.04, 0.05]	0.05
subversive	2	political foreign certain military	0.20	[0.19, 0.20]	0.06
summit	7	conference forum event meetings	0.18	[0.17, 0.18]	0.01
summit	1	first final peace general	0.13	[0.13, 0.13]	0.34
summit	2	top time end table	-0.05	[-0.06, -0.05]	0.02
target	0	targeting focus provide kill	0.19	[0.19, 0.19]	0.05
target	1	targets point top gun	0.05	[0.04, 0.05]	0.17
tending	0	designed necessary intended trying	0.20	[0.18, 0.21]	0.01
threshold	2	point end beginning eve	0.05	[nan, nan]	0.08
threshold	6	limit requirement amount ceiling	0.22	[0.21, 0.22]	0.01
threshold	1	edge brink verge line	0.12	[0.11, 0.12]	0.10

thrust	5	purpose point effect objective	0.08	[0.08, 0.08]	0.04
thrust	0	put forced imposed placed	0.01	[0.00, 0.01]	0.05
tip	6	end top edge bottom	0.16	[0.16, 0.17]	0.12
tip	0	back away together upon	0.11	[0.10, 0.11]	0.40
usage	0	law laws practice custom	0.33	[0.33, 0.34]	0.01
usage	5	constitution ordinance fashion precedent	0.62	[0.62, 0.63]	0.10
usage	2	use consumption production uses	0.34	[0.34, 0.35]	0.01
validation	0	use application recognition sale	-0.31	[-0.94, 0.31]	0.28
wired	7	called told asked contacted	0.05	[0.04, 0.05]	0.15
workshop	3	program conference meeting project	0.06	[0.05, 0.07]	0.11
workshop	1	house mill room shop	0.20	[0.19, 0.20]	0.03
workshops	7	programs meetings conferences classes	0.00	[-0.00, 0.01]	0.02
workshops	0	schools houses factories homes	0.15	[0.14, 0.15]	0.02

Usage Change Over Time

Figures S3-S8 show word usage change over time, for a random sample of 6 of the words and their respective word senses that were retained after the filtering step mentioned above. Each plot shows the proportion of word replacements belonging to a given replacement cluster, which we take to be the probability of the word being used in the sense represented by the cluster. See the GitHub repository for this paper for all remaining plots of word usage change over time. The lines in the plots are each GAM smooths (with uncertainty bands showing 95% C.I.s) calculated over the word replacement cluster data, without taking into account any speaker information. Similar plots for every single word and cluster—including both low frequency clusters as well as higher frequency clusters that did not show adequate change over time—can also be found in the GitHub repository.

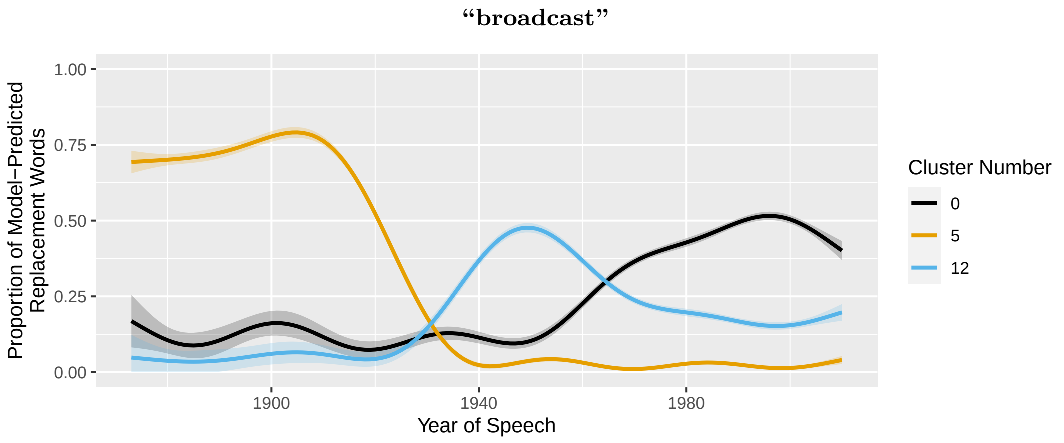

Fig. S3. “broadcast”

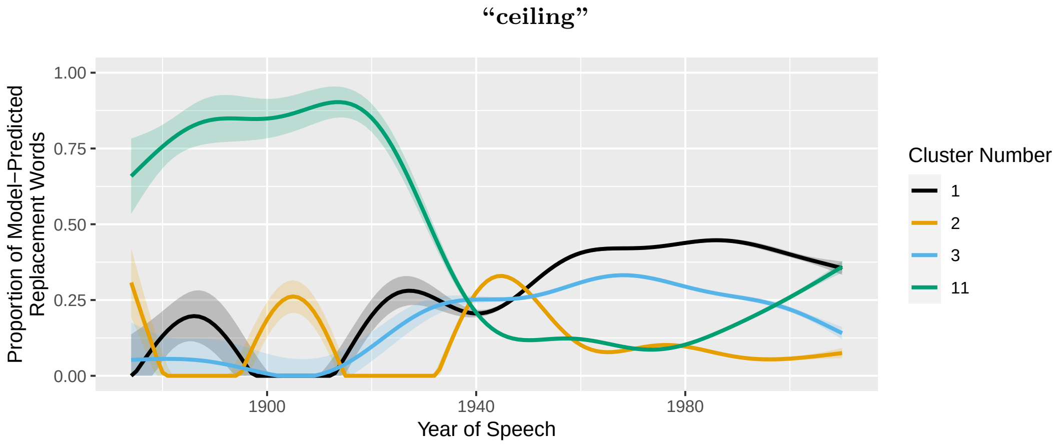

Fig. S4. “ceiling”

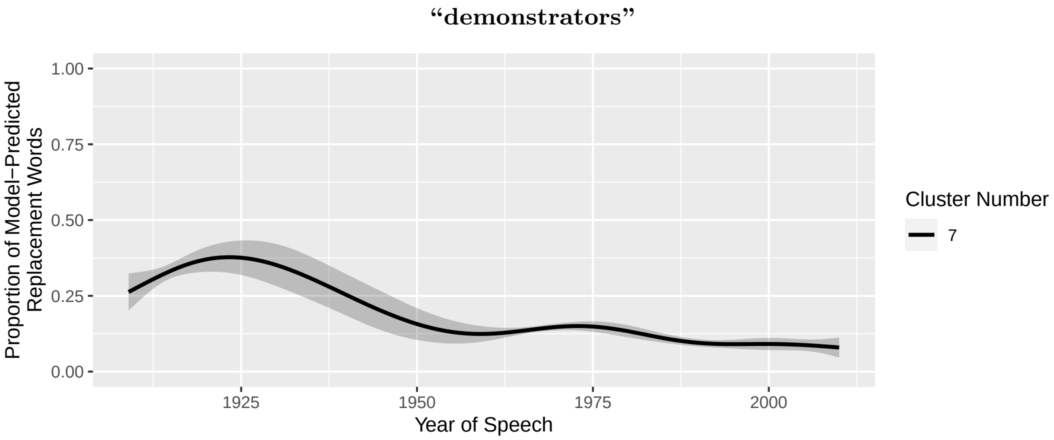

Fig. S5. “demonstrators”

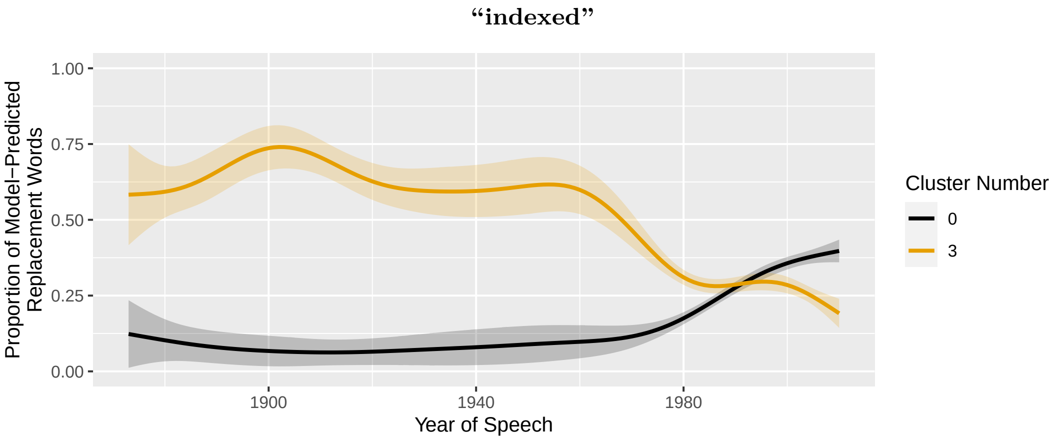

Fig. S6. “indexed”

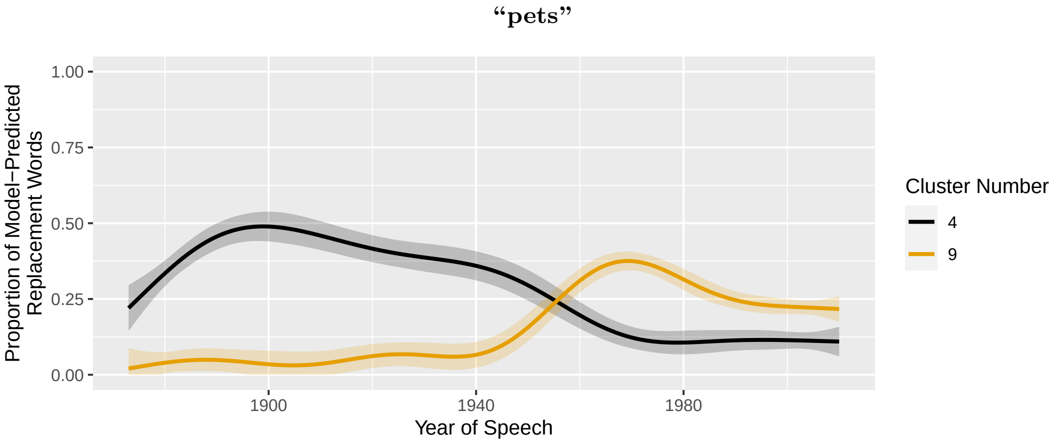

Fig. S7. “pets”

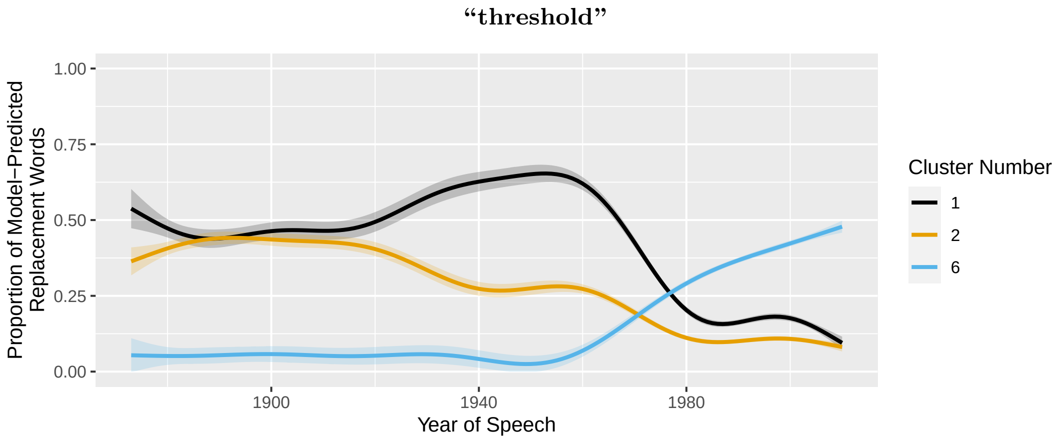

Fig. S8. “threshold”

Generalized Additive Mixed Models

Model-Fitting Implementation. We fit all GAMMs in `R`, using the `mgcv` package (3, 4). Figure S9 shows the formulas used to fit both our age effect term GAMMs (i.e. those with α, the explicit age offset term) and their unconstrained equivalents. Note that this code assumes that all previous steps of data processing—filtering, target word replacement generation, replacement clustering, and word sense probability estimation—have already been run. The code for all of these steps is available in the GitHub repository.

Age Effect Term GAMM:
```
model <- bam(cbind(n_cluster, n - n_cluster) ~ s(time) + s(speakerXsessionid, bs='re'),
            family = binomial, data = word_sense_indexed, discrete = TRUE)
```
Unconstrained GAMM:
```
model <- bam(cbind(n_cluster, n - n_cluster) ~ ti(age) + ti(year) + ti(age, year) +
             s(speakerXsessionid, bs = 're'), family = binomial, data = word_sense_indexed,
            discrete = TRUE)
```

Fig. S9. `R` code used to fit GAMMs.

In these equations, `n_cluster` is the number of replacements belonging to the word sense cluster; `n` is the total number of word replacements; `year` is the year a speech was given; `age` is the speaker's age at the time of their speech; `time` in the age effect term GAMM is defined as `year` $-$ ($\alpha *$ `age`); `speakerXsessionid` is a speaker's by-session ID (e.g. Ted Kennedy's `speakerXsessionid` is `99114941` in the 99th session of Congress, and `100114941` in the 100th session); and `word_sense_indexed` is a dataframe containing the aforementioned word replacement data for a given word sense. All other `bam` model fitting default parameters (`mgcv` version 1.9-0) are preserved.

Two of our statistical modelling choices merit discussion. The first is our choice of binomial as opposed to multiomial GAMMs, which would in principle be better-suited to this type of data. We specifically wanted to estimate an age effect per sense, as opposed to a shared age effect for all senses of a word (e.g. some senses may be uniformly adopted by speakers of all ages, while others are not). However, jointly optimizing alpha values within a multinomial GAMM was computationally infeasible using `mgcv` at the scale of our data (such models can only be fit with the slower `gam()` function rather than `bam()`).

The second is our choice of random effect structure. The variable `speakerXsessionid` *jointly* encodes both speaker and session. While this allows for by-speaker and by-session variability, it does not model higher-order dependencies, such as those between the same speaker in different sessions. Other possible random effect structures were not used for practical or conceptual reasons: using random effect terms for speaker identity *without* encoding session variability led to poorer model fit; adding independent random effect terms for both speaker and session would miss key interactions; and adding *both* independent and interaction terms between the two proved computationally infeasible. We chose joint speaker-session identifiers as a compromise between model fit and computational cost.

α-Optimization Details. Figure S10 shows the code we use to optimize for α values by using maximum likelihood estimation (using `mle()` from the `stats4` package in `R` (4)). See Table S1 for the full set of (GAMM-derived) α-estimates for all word senses modelled.

As detailed in *Materials and Methods* in the main paper, due to the computational cost of optimization, we optimize over only a subset of data for some senses, taking speeches from the most prolific speakers by session for each word. In order to do so, for each target word, we iteratively exclude speeches from speaker-session pairs attached to only $1, \ldots, n$ speeches containing the target word, until we are left with speeches from 4000 or fewer unique speaker-session combinations. In practice, this often

```
compute_fREML <- function(a, data) {
            data$time <- data$year - (a * data$age)
            model <- bam(cbind(n_cluster, n - n_cluster) ~ s(time) + s(speakerXsessionid, bs='re'),
                        family = binomial, data = data, discrete = TRUE)
            model_summary <- summary(model, re.test=FALSE)
            # Return the fREML value:
            return(as.numeric(model_summary$sp.criterion))
            }

negative_log_likelihood <- function(a){
            compute_fREML(a, data = filtered_data)
            }
mle_result <- mle(minuslogl = negative_log_likelihood, start = list(a = starting_point), method = "Brent",
            lower = lower_bound, upper = upper_bound)
```

Fig. S10. R code used to estimate α-values.

means only excluding data from speakers who only use the target word once in a session, or in cases involving fewer than 4000 speaker-session pairs to begin with, zero data loss; all code used to implement this filtering step is included in the GitHub repository.

Table S2 shows, for each word, the amount of data lost in the filtering process before α-optimization. Note, however, that this only applies to the step of *finding* an optimal α-value. When we actually fit our age effect term GAMMs, we do so using all of the data available for a given word.

Table S2. Data size when optimizing for α, and fitting GAMMs. For fitting GAMMs, the total number of speeches containing each target word was used; for estimating α-values, a smaller subset was used.

Word	Total No. of Speeches	No. of Speeches Used for α-Optimization	Proportion of Data Ignored During α-Optimization
awful	13,999	10,010	0.28
broadcast	11,745	7,956	0.32
monitor	14,641	11,479	0.22
guy	9,532	6,838	0.28
headed	23,777	14,506	0.39
major	221,008	145,881	0.34
plane	20,171	15,065	0.25
tip	9,765	5,901	0.4
graft	2,635	2,635	0
outstanding	90,976	58,236	0.36
astronomical	3,395	3,395	0
ceiling	28,255	21,578	0.24
thrust	17,712	13,631	0.23
challenges	32,798	25,825	0.21
articles	61,268	40,866	0.33
dial	10,828	8,376	0.23
recession	24,936	21,970	0.12
arraignment	986	986	0
receiver	3,754	3,754	0
maneuvering	2,291	2,291	0
target	29,614	22,309	0.25
segregation	7,791	7,791	0
lunatic	926	926	0
rendezvous	992	992	0
pregnant	7,338	7,338	0
implicit	6,735	6,735	0
retarded	4,331	4,331	0
receivers	3,946	3,946	0
locator	472	472	0
alienation	1,677	1,677	0
demonstrators	2,047	2,047	0
usage	7,761	4,483	0.42
appraising	860	860	0

cutoff	5,326	5,326	0
subversive	6,396	6,396	0
forgiveness	3,269	3,269	0
folders	275	275	0
workshops	2,584	2,584	0
drives	6,584	3,501	0.47
summit	11,032	8,636	0.22
background	32,354	23,650	0.27
stewards	2,133	2,133	0
organ	6,859	3,611	0.47
tending	3,929	3,929	0
needle	1,761	1,761	0
coaches	2,272	2,272	0
particle	4,473	4,473	0
sterling	5,434	5,434	0
fixation	694	694	0
cap	31,341	21,534	0.31
masters	9,351	5,349	0.43
indexed	1,892	1,892	0
powerhouse	750	750	0
forensic	1,035	1,035	0
satellite	10,298	7,841	0.24
clearance	6,133	6,133	0
innovation	13,126	9,960	0.24
validation	713	713	0
match	17,874	13,138	0.26
hybrid	2,188	2,188	0
package	54,085	42,795	0.21
concerns	109,108	75,149	0.31
fabulous	2,292	2,292	0
sector	51,114	39,135	0.23
shark	714	714	0
caps	8,311	8,311	0
glue	937	937	0
threshold	10,514	7,260	0.31
amorphous	487	487	0
substance	46,406	30,250	0.35
signal	24,320	16,039	0.34
promiscuous	476	476	0
extremity	1,172	1,172	0
fantastic	6,298	6,298	0
pets	1,086	1,086	0
projection	4,393	4,393	0
wired	2,050	2,050	0
workshop	1,845	1,845	0
shorthand	838	838	0
platforms	5,679	5,679	0
fans	3,224	3,224	0

Figure S11 also shows, for our α estimates, the distribution of 95% confidence interval widths by total data size (measured in terms of log word frequency). As it indicates, the wider confidence intervals in our data (see Figure 5 in the main paper) are generated from words with low frequencies across the corpus.

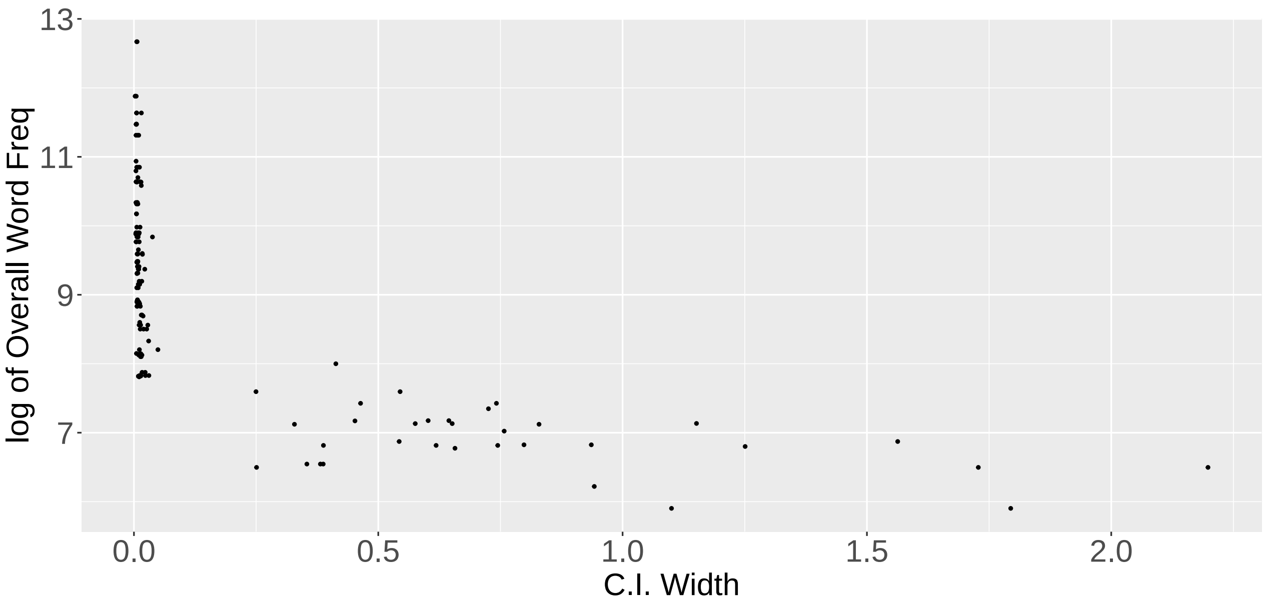

Fig. S11. Scatterplot showing the 95% confidence interval widths of our α estimates (| upper bound − lower bound |), against the natural log of the overall frequency of the associated word across the entire corpus. As the plot indicates, words with lower amounts of data generate more uncertain estimates (i.e., wider confidence intervals).

GAMM Prediction Plots. Figures S14-S45 show prediction heatmaps from a random sample of 32 of the 163 word senses we model. We present the predictions from our age effect term GAMMs alongside those from our unconstrained GAMMs; all remaining heatmaps are included in our GitHub repository.

Goodness of Fit of Age Effect Term GAMMs. To assess how well our explicit age effect term GAMMs fit the data, we compare them with the more unconstrained GAMMs that model word sense probability as *any* joint function of age and speech year (see *Modelling Methods* in the body of the main paper, as well as *Materials and Methods*). We find that the two produce almost identical adjusted R-squared metrics, which can be found in full in the paper's GitHub repository. However, the degree to which this indicates our α-estimate is a reliable approximation of the data is unclear—much of this variance may be accounted for by to the models' random effects, instead of the age effect term. We therefore instead evaluate the α-based GAMMs against the unconstrained GAMMs; if the former are indeed a good model of the underlying data, the two approaches should yield similar results.

For each pair of α-based and unconstrained GAMMs, we compute the L^2 Distance between their full sets of predictions for an average speaker-session pair, normalized by the L^2 Distance between the predictions from unconstrained GAMM and the mean of all its predictions for the same average speaker-session pair. This can be described by the following equation:

$$\Delta(w) = \frac{\sum_{i=1}^{n}(U_i^w - V_i^w)^2}{\sum_{i=1}^{n}(U_i^w - \bar{U^w})^2}$$

Where w is some word sense, n is the number of valid speaker-session pairs, U^w is the full vector of predictions given an average speaker-session pair from the unconstrained GAMM fitted to model the probability of w, V^w is the full vector of predictions given the same speaker-session pair from the corresponding explicit age effect GAMM, and $\bar{U^w}$ is the mean of all predictions in U^w. The lower this value is, the closer the two models' full sets of predictions are, relative to the amount of variation in the predictions, indicating that the α-based GAMM is a good approximation of the unconstrained GAMM. Table S1 includes the full set of normalized L^2 Distance values; these are also indicated in Figures S14-S45. For the vast majority of word senses, these values are quite low, indicating that the age term GAMMs are a good model of the underlying structure in the data.

Bayesian Meta-Analysis Model

We fit our Bayesian meta-analysis model of α-values using the the `brms` front-end (5) to Stan (6). The relevant code used to fit this model is provided in Figure S12 below, along with the full fitted model summary in Figure S13.

```
library(brms)
collated <- read_csv("collated_results.csv")
collated <- collated %>% mutate(std_err = (a_estimate-ci_lower)/1.96,
                        sq_error = (mean(a_estimate) - a_estimate)^2,
                        log_word_freq = log(overall_word_freq),
                        word = as.factor(word),
                        word_sense = as.factor(paste(word, as.character(sense))))
# Fitting Bayesian meta-analysis model:
priors <- c(prior(normal(0, 1), class = Intercept),
                        prior(normal(0, 1), class = sd))
fit_ma <- brm(a_estimate | resp_se(std_err, sigma = FALSE) ~
                        1 + (1 | word_sense),
                        data = collated,
                        prior = priors,
                        control = list(adapt_delta = .99, max_treedepth = 20),
                        iter = 4000,
                        file = "meta-analysis.rds")
```

Fig. S12. R code used to fit Bayesian meta-analysis model.

```
> summary(fit_ma)
 Family:  gaussian
 Links:  mu = identity; sigma = identity
Formula:  a_estimate | resp_se(std_err, sigma = FALSE) ~ 1 + (1 | word_sense)
 Data:  collated (Number of observations:  162)
 Draws:  4 chains, each with iter = 4000; warmup = 2000; thin = 1;
 total post-warmup draws = 8000

Group-Level Effects:
 word_sense (Number of levels:  163)
              Estimate  Est.Error  l-95% CI  u-95% CI  Rhat  Bulk_ESS  Tail_ESS
 sd(Intercept)    0.18      0.01      0.16      0.20  1.01      398       710


Population-Level Effects:
           Estimate  Est.Error  l-95% CI  u-95% CI  Rhat  Bulk_ESS  Tail_ESS
 Intercept    0.13      0.01      0.10      0.16  1.03      162       330

Family Specific Parameters:
      Estimate  Est.Error  l-95% CI  u-95% CI  Rhat  Bulk_ESS  Tail_ESS
 sigma    0.00      0.00      0.00      0.00    NA       NA        NA

Draws were sampled using sampling(NUTS). For each parameter, Bulk_ESS
and Tail_ESS are effective sample size measures, and Rhat is the potential
scale reduction factor on split chains (at convergence, Rhat = 1).
```

Fig. S13. Full summary of Bayesian meta-analysis model. Here, the population-level intercept, which corresponds to the estimated average age effect , is of primary interest.

Speaker- and Generation-wise Usage Change

Figures S46-S61 show word sense usage change at a speaker-wise level and at a generation-wise level. Data sparsity meant this analysis was not possible for every word sense fitted. Instead, we consider one sense for each of the eight most frequently-used target words in the corpus. Note that parts of uncertainty bands are not plotted in some of these figures for years when there is high data sparsity.

“indexed”, sense 3

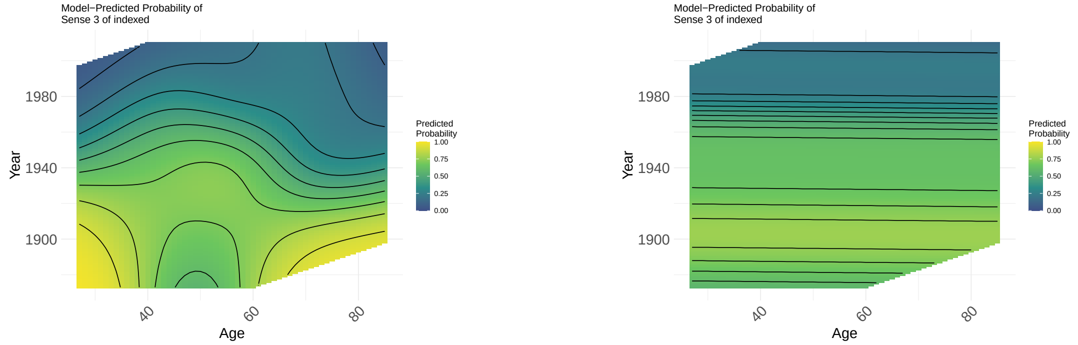

(a) Unconstrained GAMM

(b) Age Effect Term GAMM

Fig. S14. “indexed”, sense 3. Normalized L2 Distance: 0.26

“threshold”, sense 1

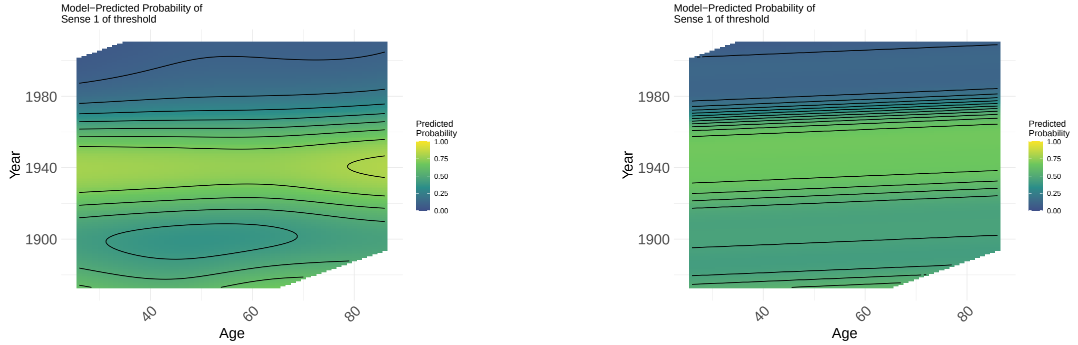

(a) Unconstrained GAMM

(b) Age Effect Term GAMM

Fig. S15. “threshold”, sense 1. Normalized L2 Distance: 0.10

“pets”, sense 9

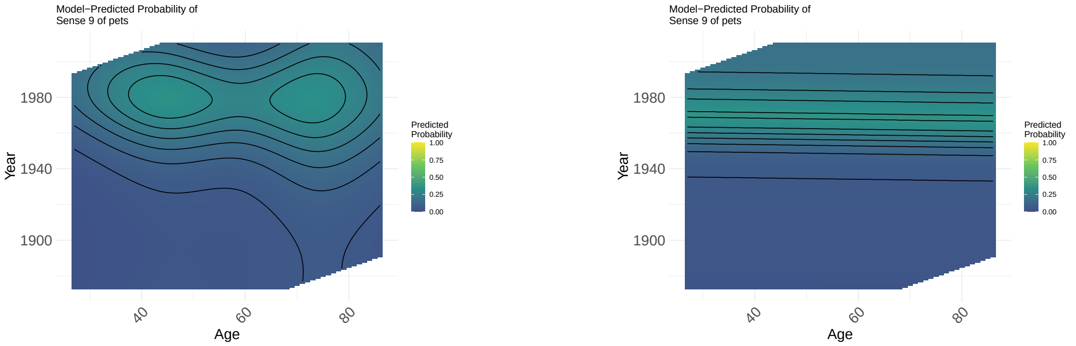

(a) Unconstrained GAMM

(b) Age Effect Term GAMM

Fig. S16. “pets”, sense 9. Normalized L2 Distance: 0.22

“demonstrators”, sense 7

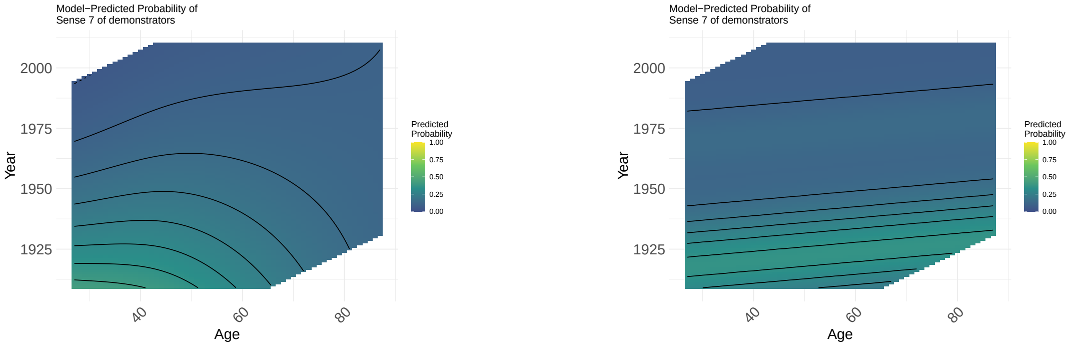

(a) Unconstrained GAMM

(b) Age Effect Term GAMM

Fig. S17. “demonstrators”, sense 7. Normalized L2 Distance: 0.53

“broadcast”, sense 0

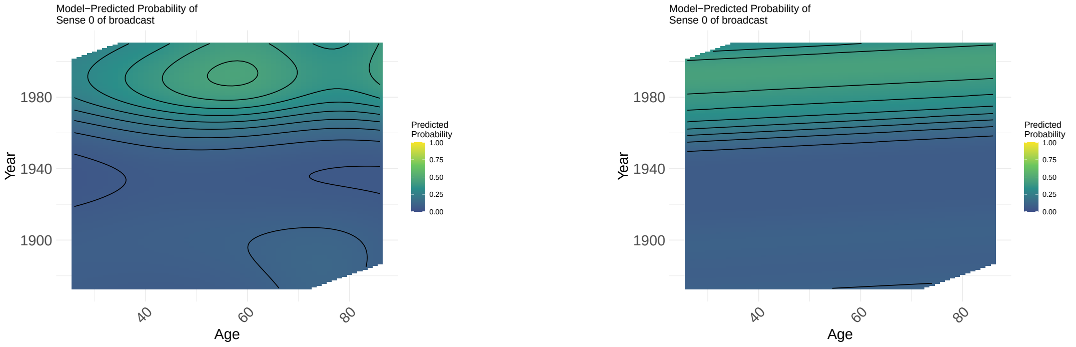

(a) Unconstrained GAMM

(b) Age Effect Term GAMM

Fig. S18. “broadcast”, sense 0. Normalized L2 Distance: 0.07

“ceiling”, sense 2

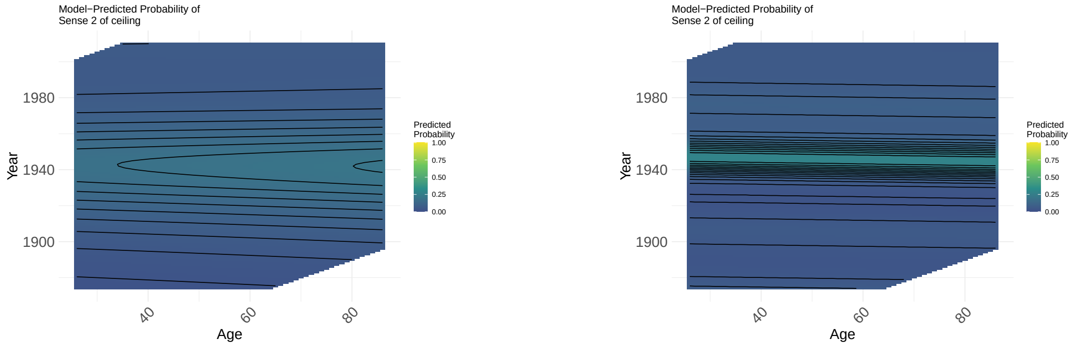

(a) Unconstrained GAMM **(b)** Age Effect Term GAMM

Fig. S19. “ceiling”, sense 2. Normalized L2 Distance: 1.03

“satellite”, sense 5

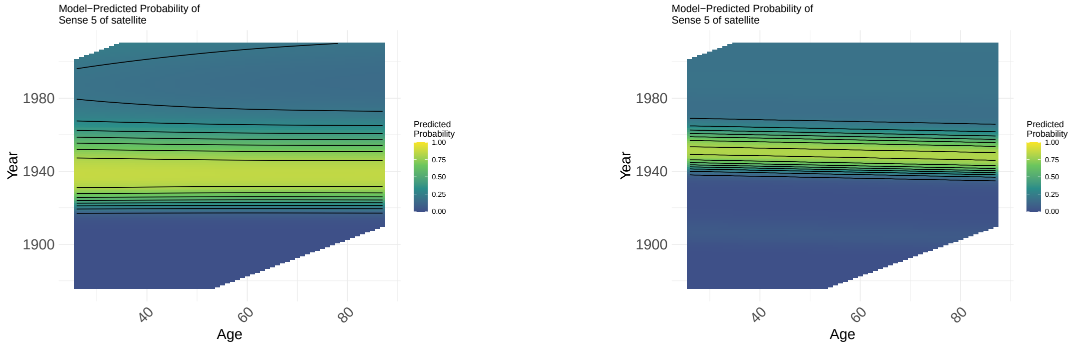

(a) Unconstrained GAMM **(b)** Age Effect Term GAMM

Fig. S20. “satellite”, sense 5. Normalized L2 Distance: 0.88

“concerns”, sense 0

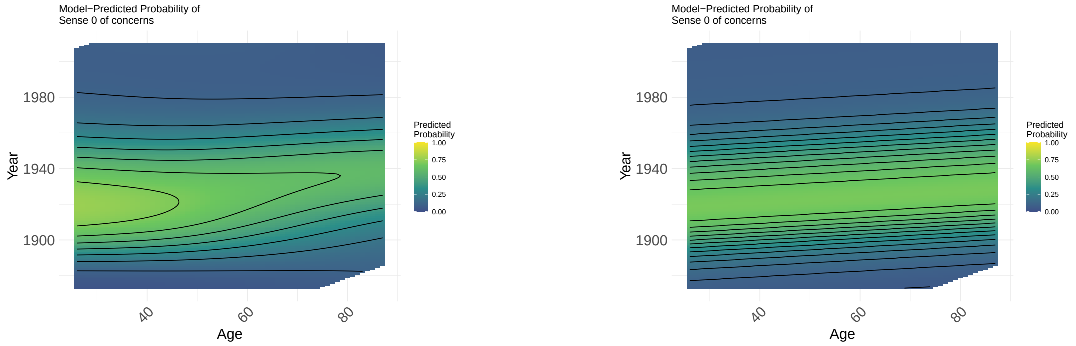

(a) Unconstrained GAMM **(b)** Age Effect Term GAMM

Fig. S21. “concerns”, sense 0. Normalized L2 Distance: 0.05

“rendezvous”, sense 1

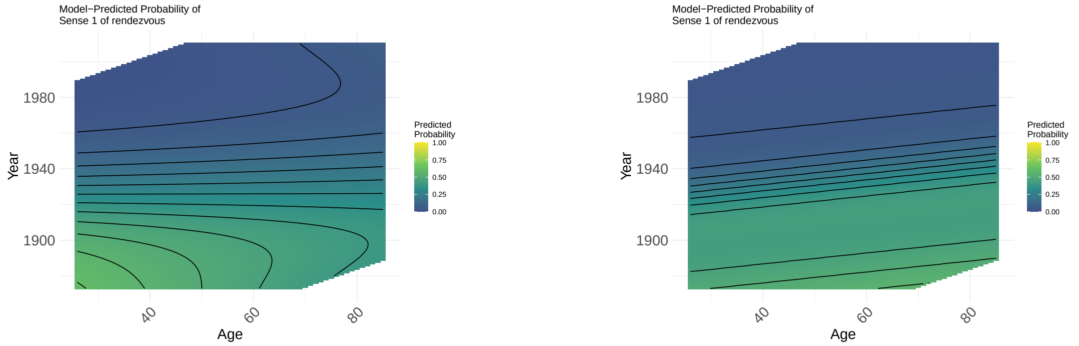

(a) Unconstrained GAMM

(b) Age Effect Term GAMM

Fig. S22. “rendezvous”, sense 1. Normalized L2 Distance: 0.07

“particle”, sense 4

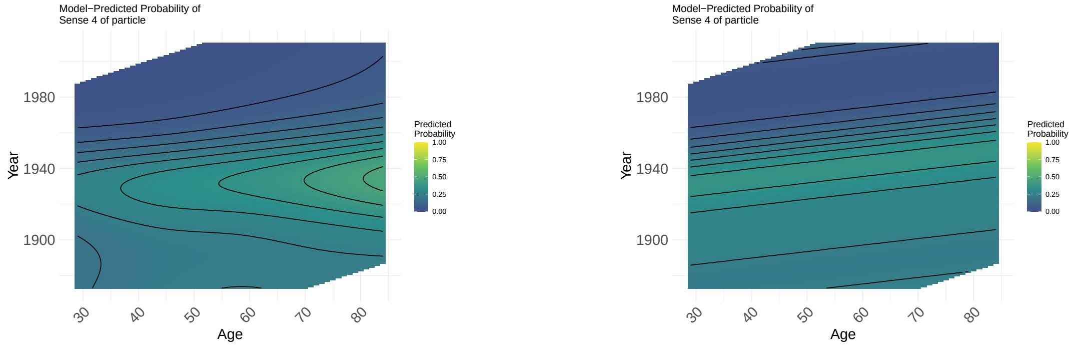

(a) Unconstrained GAMM

(b) Age Effect Term GAMM

Fig. S23. “particle”, sense 4. Normalized L2 Distance: 0.14

“arraignment”, sense 12

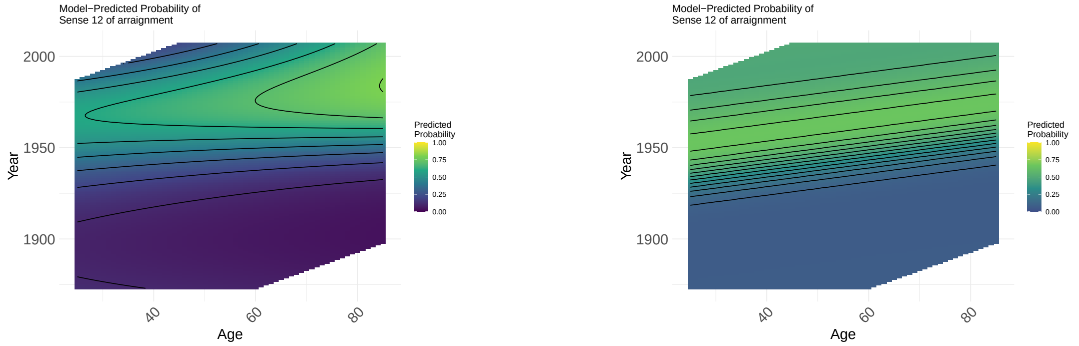

(a) Unconstrained GAMM

(b) Age Effect Term GAMM

Fig. S24. “arraignment”, sense 12. Normalized L2 Distance: 0.11

“cutoff”, sense 4

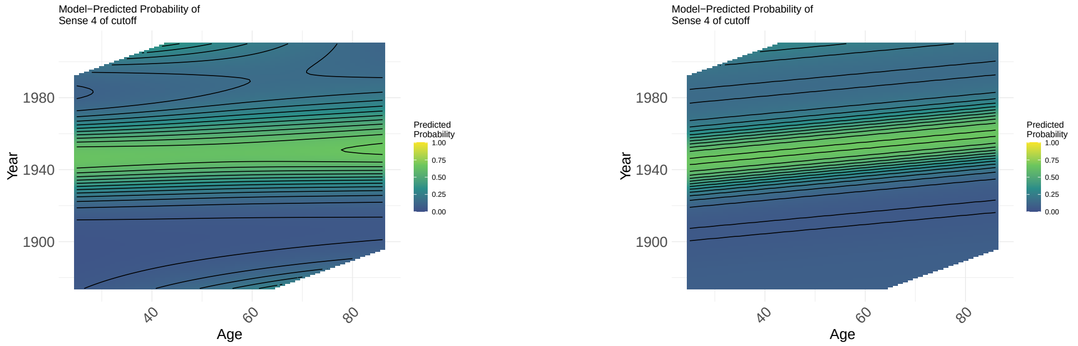

(a) Unconstrained GAMM **(b)** Age Effect Term GAMM

Fig. S25. “cutoff”, sense 4. Normalized L2 Distance: 0.13

“summit”, sense 7

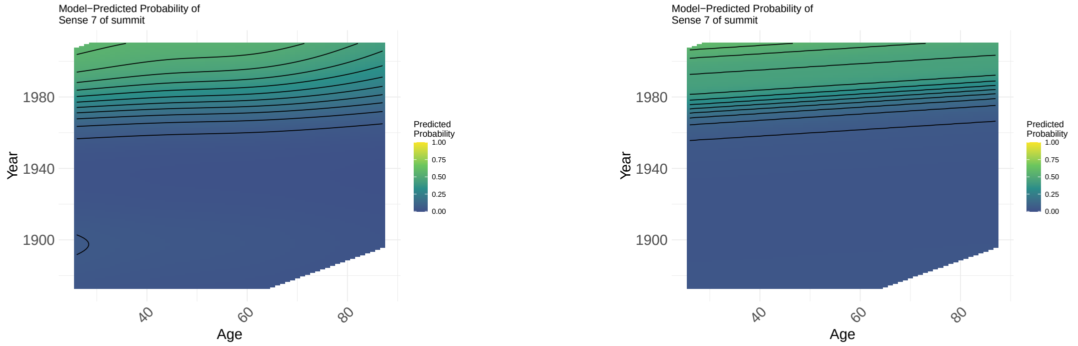

(a) Unconstrained GAMM **(b)** Age Effect Term GAMM

Fig. S26. “summit”, sense 7. Normalized L2 Distance: 0.01

“powerhouse”, sense 7

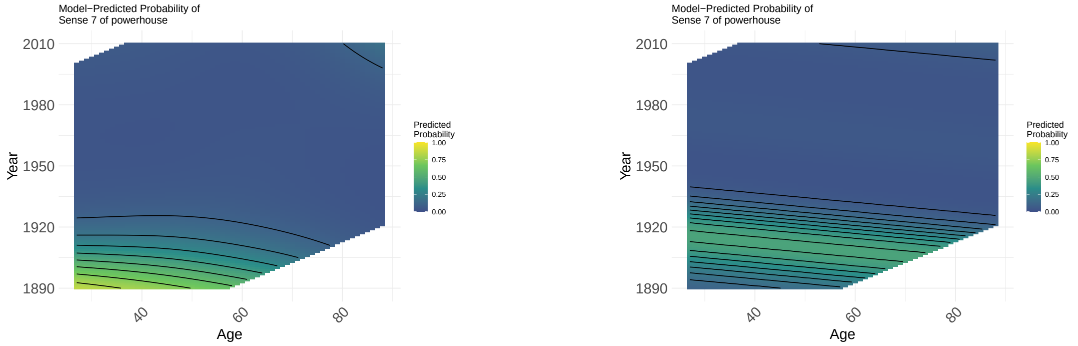

(a) Unconstrained GAMM **(b)** Age Effect Term GAMM

Fig. S27. “powerhouse”, sense 7. Normalized L2 Distance: 0.72

“match”, sense 6

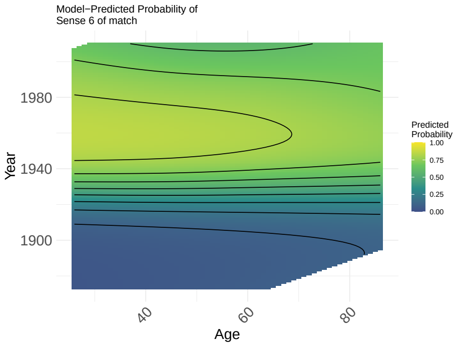

(a) Unconstrained GAMM

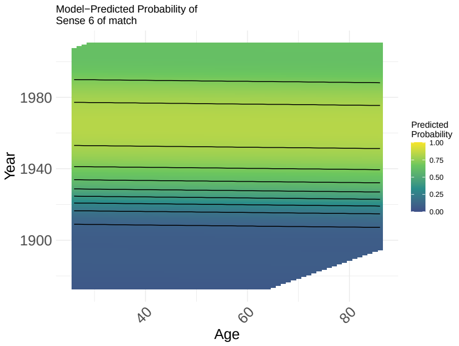

(b) Age Effect Term GAMM

Fig. S28. “match”, sense 6. Normalized L2 Distance: 0.01

“target”, sense 0

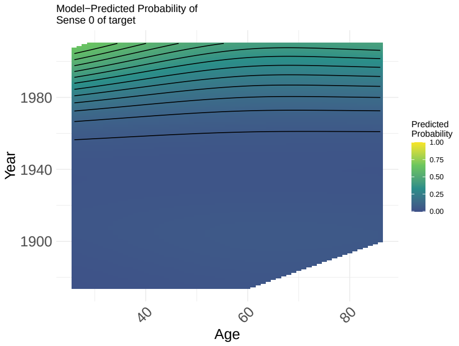

(a) Unconstrained GAMM

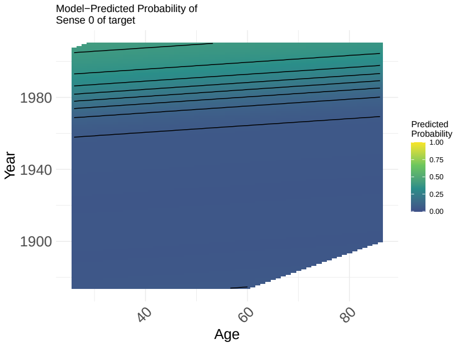

(b) Age Effect Term GAMM

Fig. S29. “target”, sense 0. Normalized L2 Distance: 0.05

“promiscuous”, sense 1

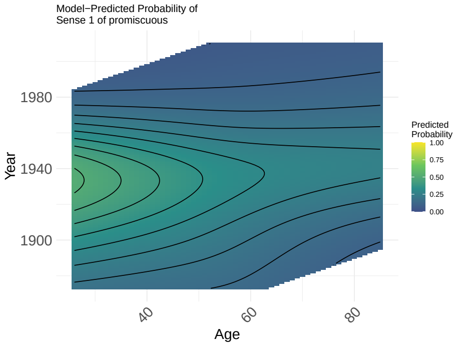

(a) Unconstrained GAMM

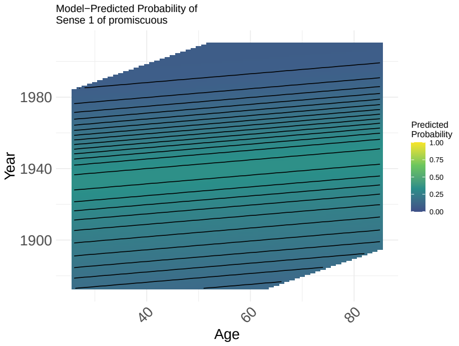

(b) Age Effect Term GAMM

Fig. S30. “promiscuous”, sense 1. Normalized L2 Distance: 0.27

“sterling”, sense 5

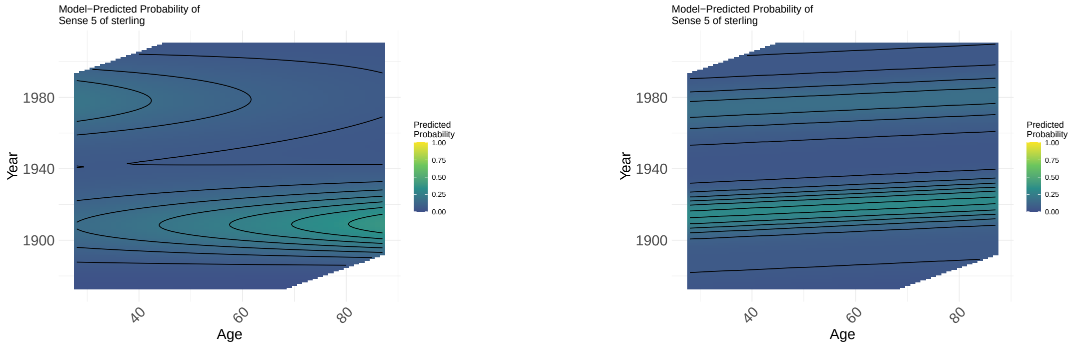

(a) Unconstrained GAMM

(b) Age Effect Term GAMM

Fig. S31. “sterling”, sense 5. Normalized L2 Distance: 0.85

“amorphous”, sense 2

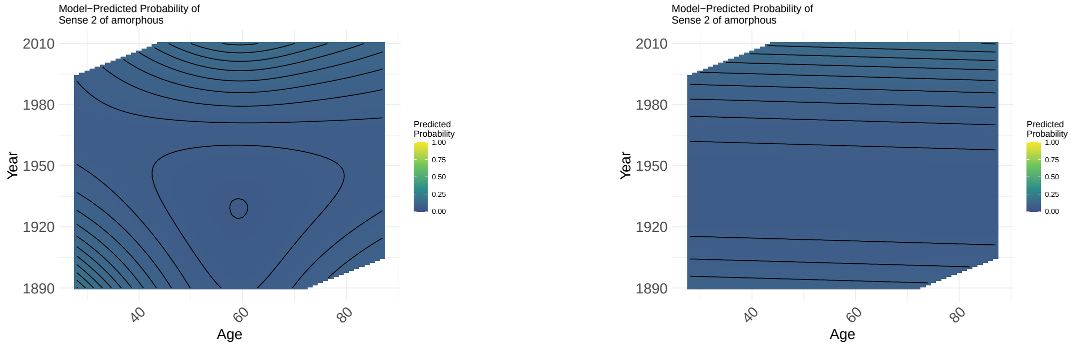

(a) Unconstrained GAMM

(b) Age Effect Term GAMM

Fig. S32. “amorphous”, sense 2. Normalized L2 Distance: 0.46

“graft”, sense 8

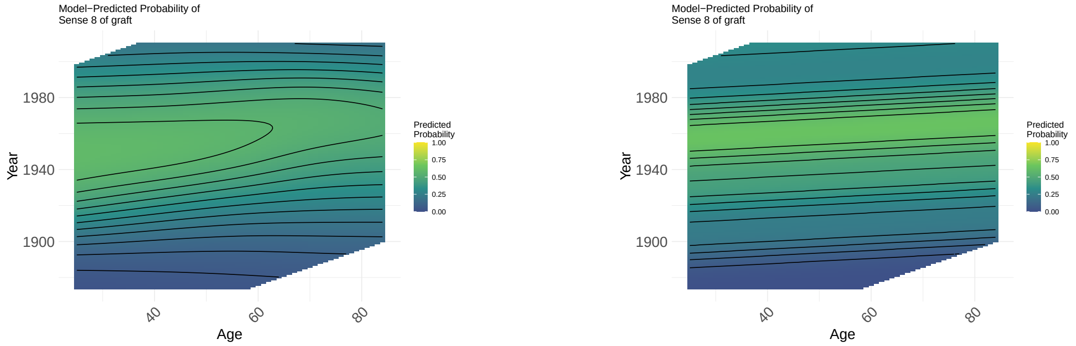

(a) Unconstrained GAMM

(b) Age Effect Term GAMM

Fig. S33. “graft”, sense 8. Normalized L2 Distance: 0.10

“thrust”, sense 0

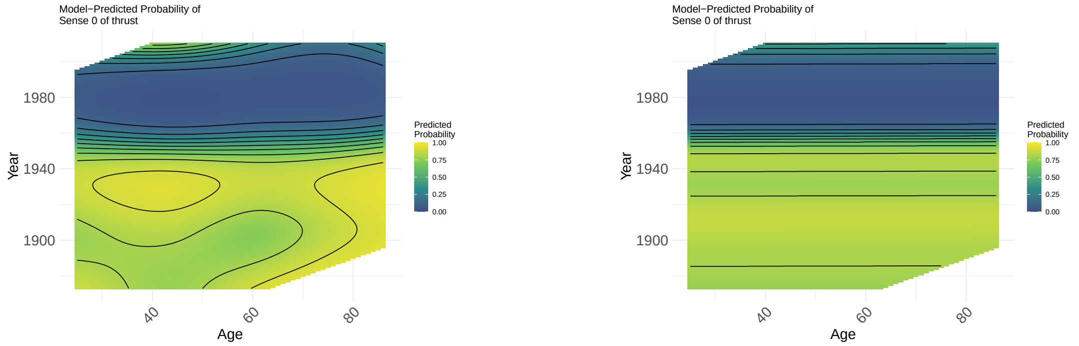

(a) Unconstrained GAMM **(b)** Age Effect Term GAMM

Fig. S34. “thrust”, sense 0. Normalized L2 Distance: 0.05

“fans”, sense 3

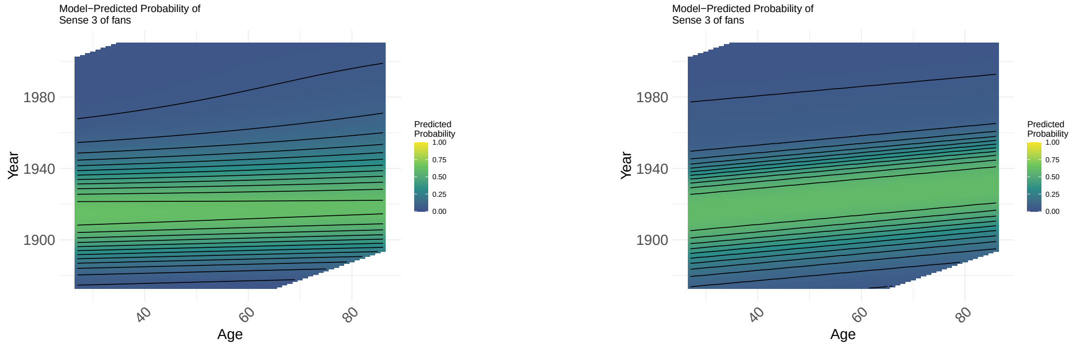

(a) Unconstrained GAMM **(b)** Age Effect Term GAMM

Fig. S35. “fans”, sense 3. Normalized L2 Distance: 0.08

“monitor”, sense 2

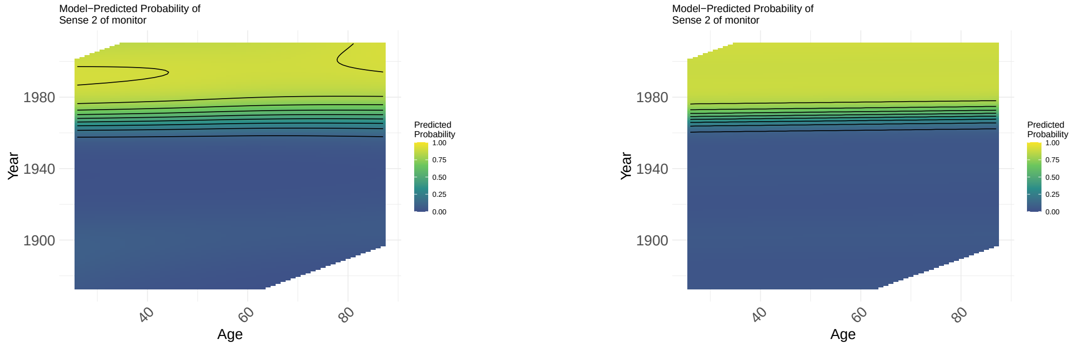

(a) Unconstrained GAMM **(b)** Age Effect Term GAMM

Fig. S36. “monitor”, sense 2. Normalized L2 Distance: 0.01

“concerns”, sense 2

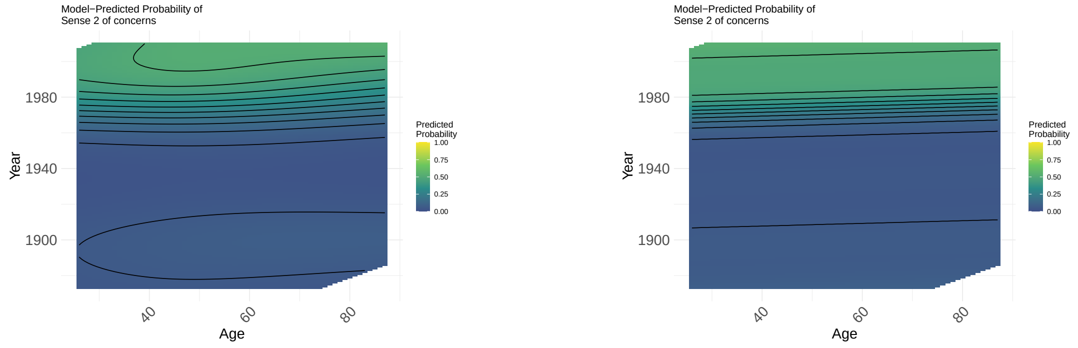

(a) Unconstrained GAMM **(b)** Age Effect Term GAMM

Fig. S37. “concerns”, sense 2. Normalized L2 Distance: 0.02

“match”, sense 9

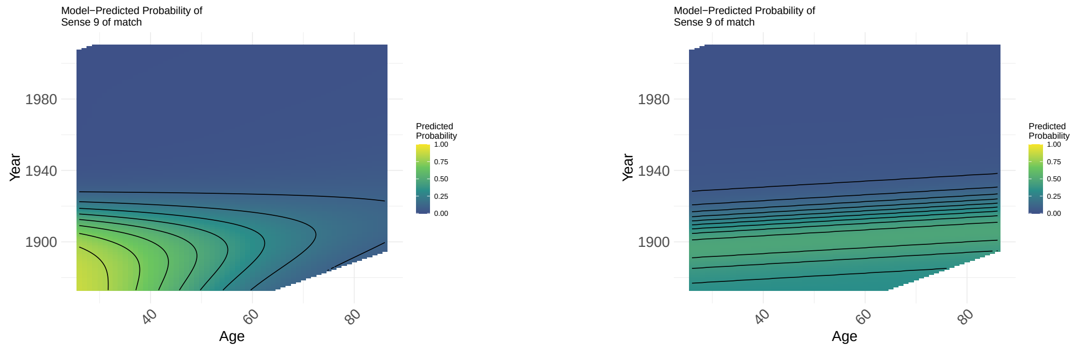

(a) Unconstrained GAMM **(b)** Age Effect Term GAMM

Fig. S38. “match”, sense 9. Normalized L2 Distance: 0.36

“usage”, sense 0

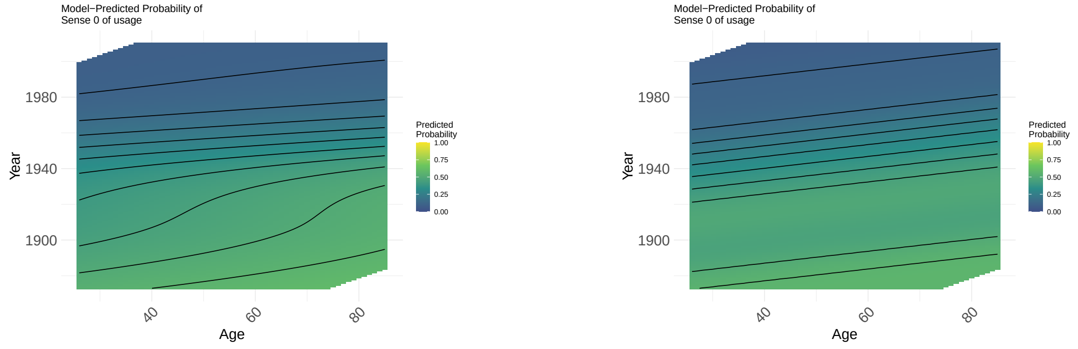

(a) Unconstrained GAMM **(b)** Age Effect Term GAMM

Fig. S39. “usage”, sense 0. Normalized L2 Distance: 0.01

“astronomical”, sense 4

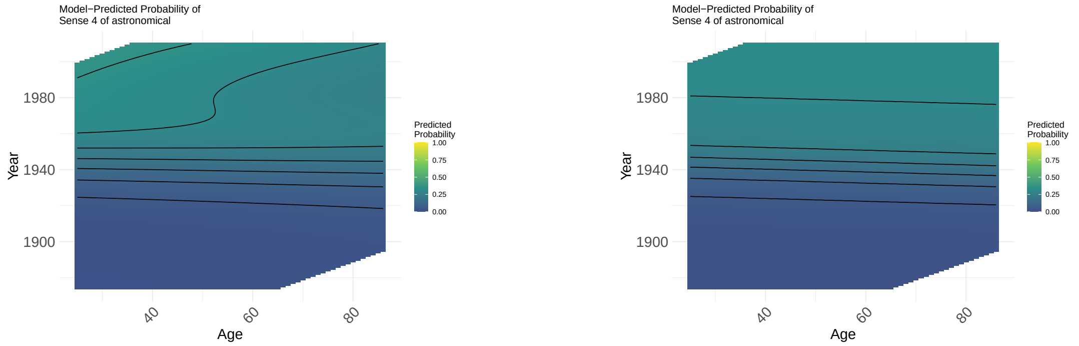

(a) Unconstrained GAMM

(b) Age Effect Term GAMM

Fig. S40. “astronomical”, sense 4. Normalized L2 Distance: 0.01

“appraising”, sense 1

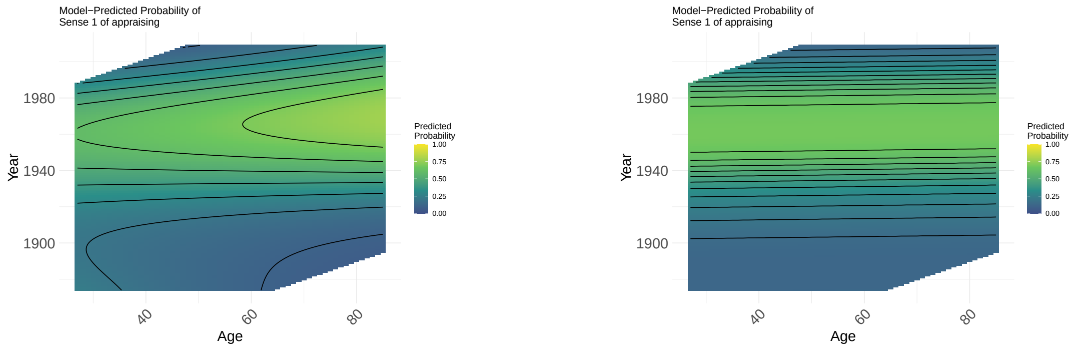

(a) Unconstrained GAMM

(b) Age Effect Term GAMM

Fig. S41. “appraising”, sense 1. Normalized L2 Distance: 0.07

“tending”, sense 0

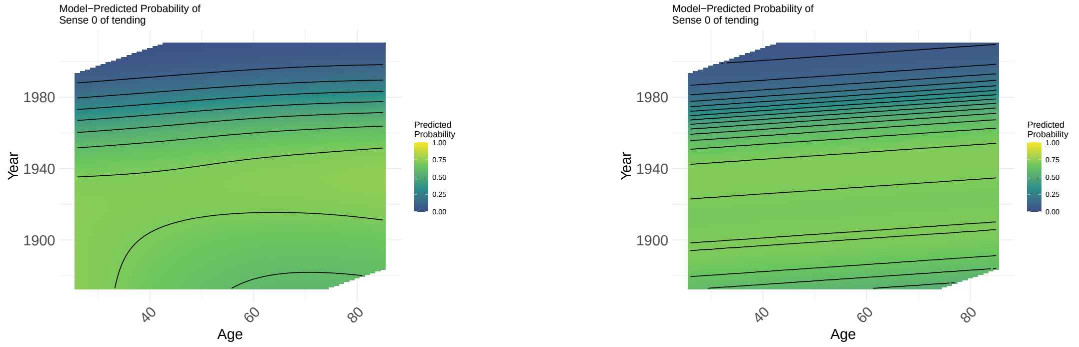

(a) Unconstrained GAMM

(b) Age Effect Term GAMM

Fig. S42. “tending”, sense 0. Normalized L2 Distance: 0.01

“segregation”, sense 2

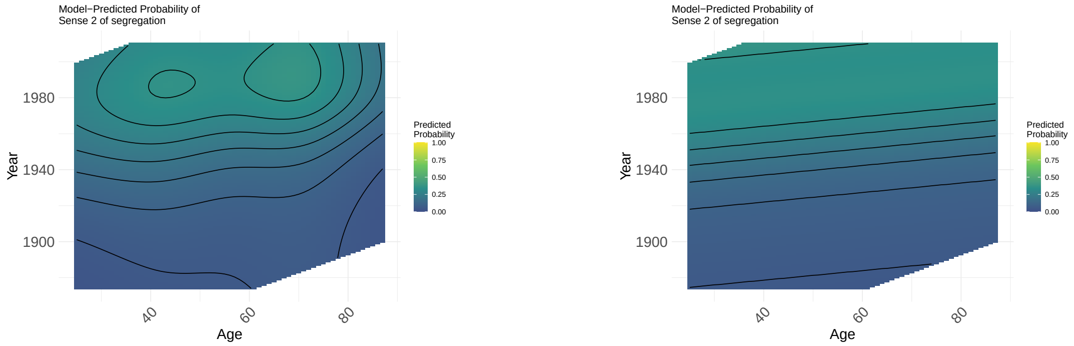

(a) Unconstrained GAMM

(b) Age Effect Term GAMM

Fig. S43. “segregation”, sense 2. Normalized L2 Distance: 0.07

“tip”, sense 6

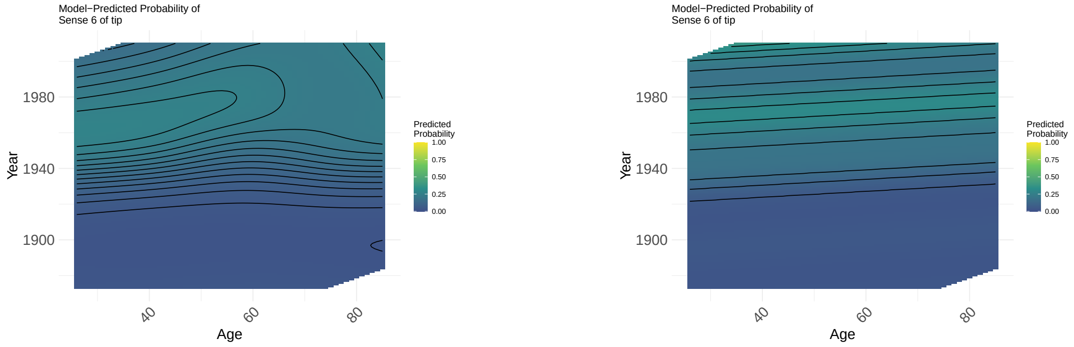

(a) Unconstrained GAMM

(b) Age Effect Term GAMM

Fig. S44. “tip”, sense 6. Normalized L2 Distance: 0.12

“organ”, sense 5

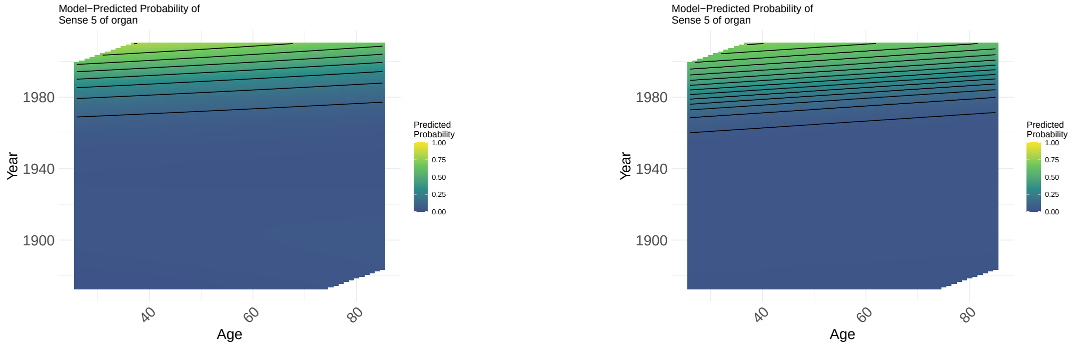

(a) Unconstrained GAMM

(b) Age Effect Term GAMM

Fig. S45. “organ”, sense 5. Normalized L2 Distance: 0.01

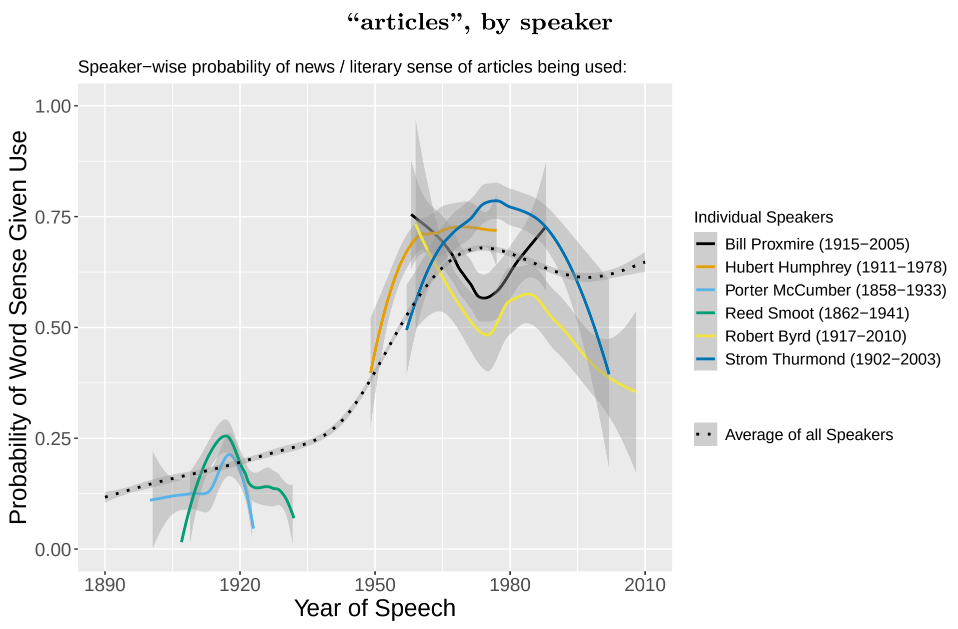

Fig. S46. "articles"

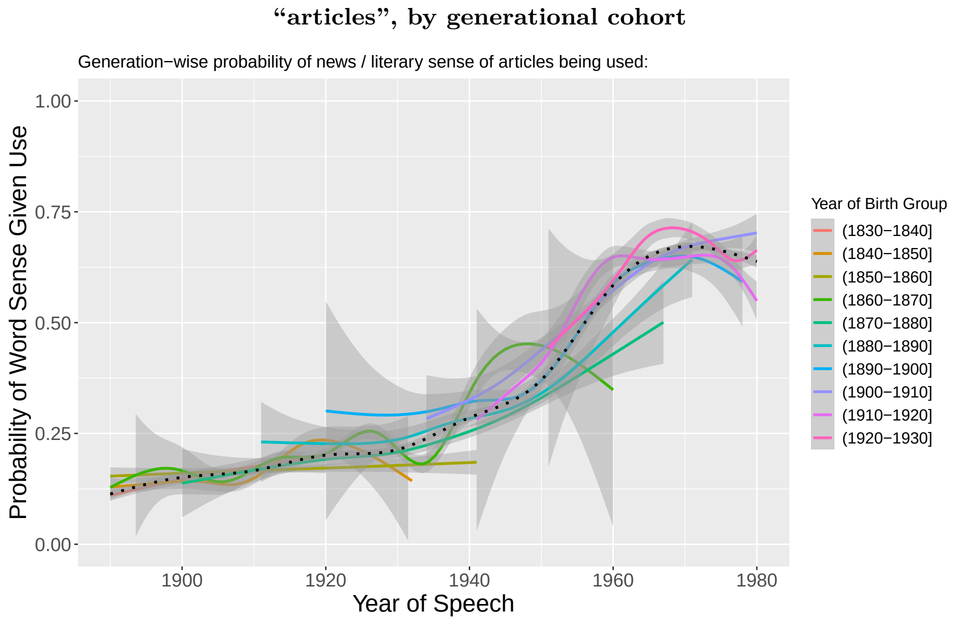

Fig. S47. "articles"

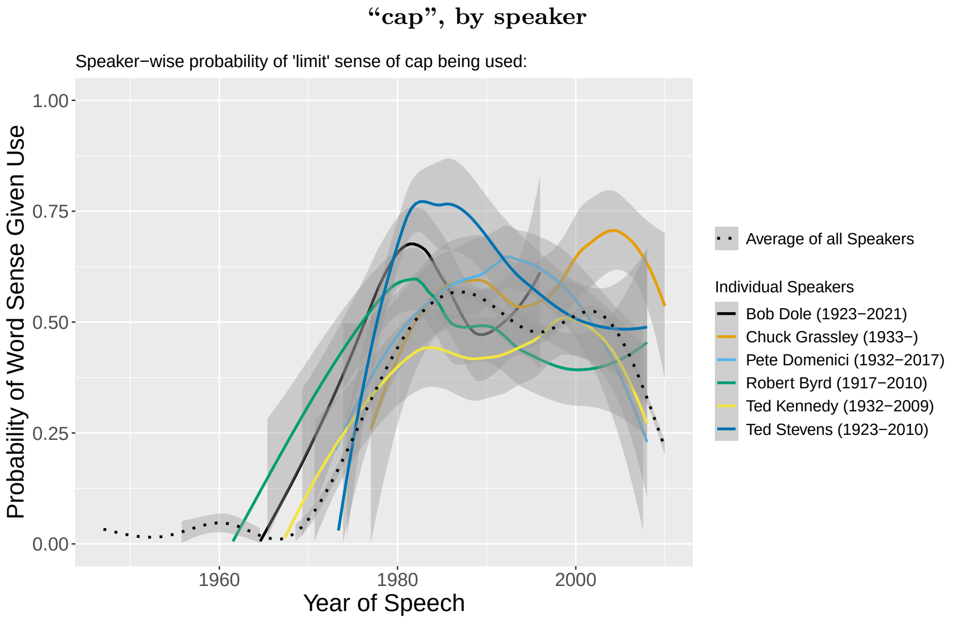

Fig. S48. “cap”

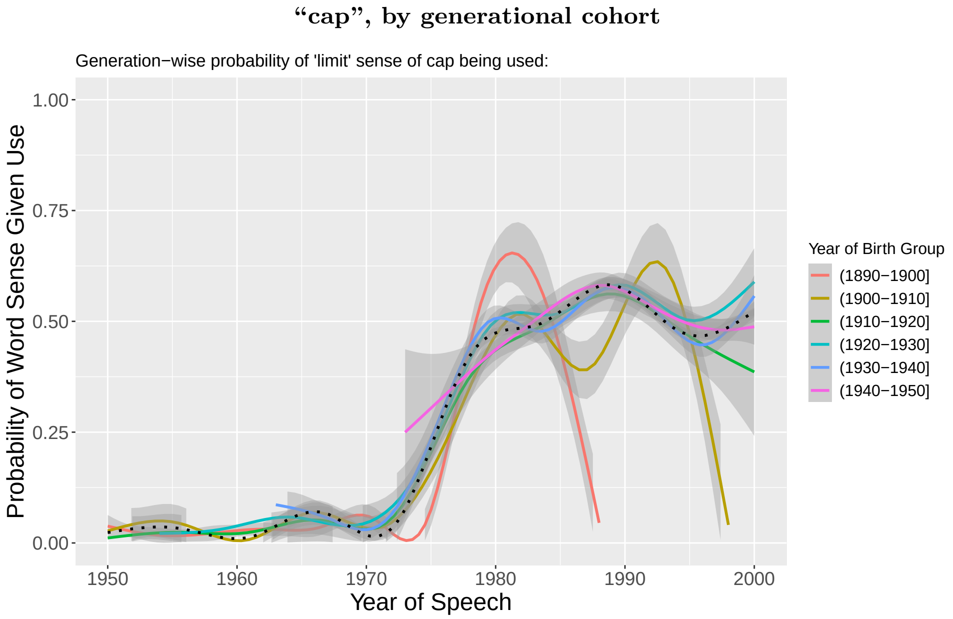

Fig. S49. “cap”

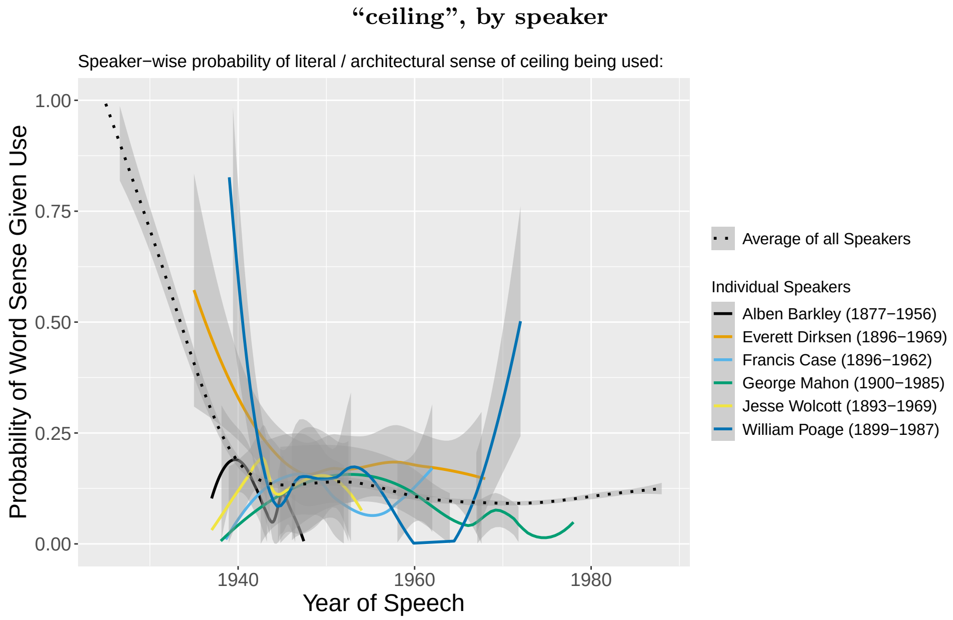

Fig. S50. “ceiling”

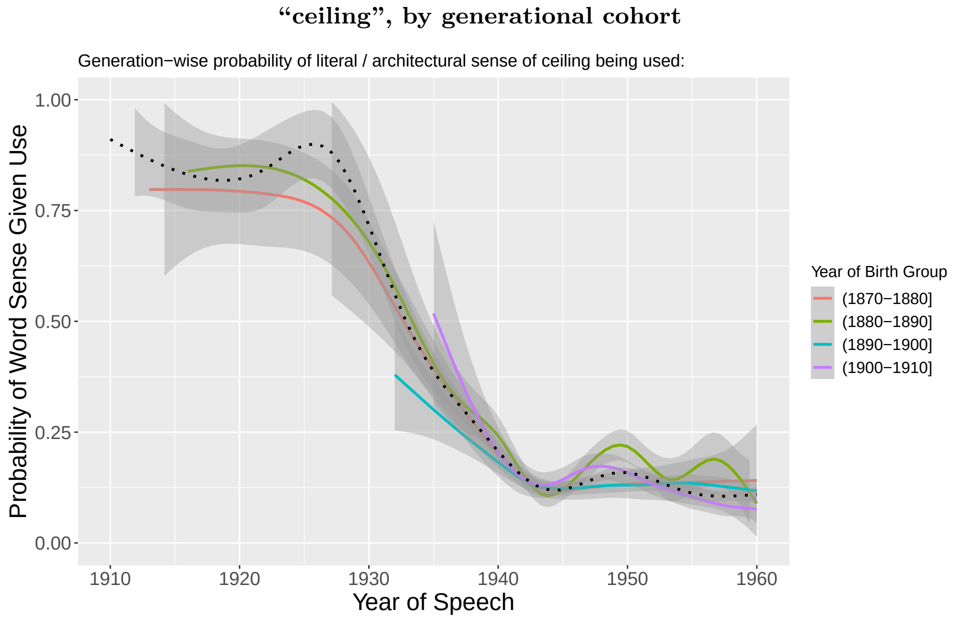

Fig. S51. “ceiling”

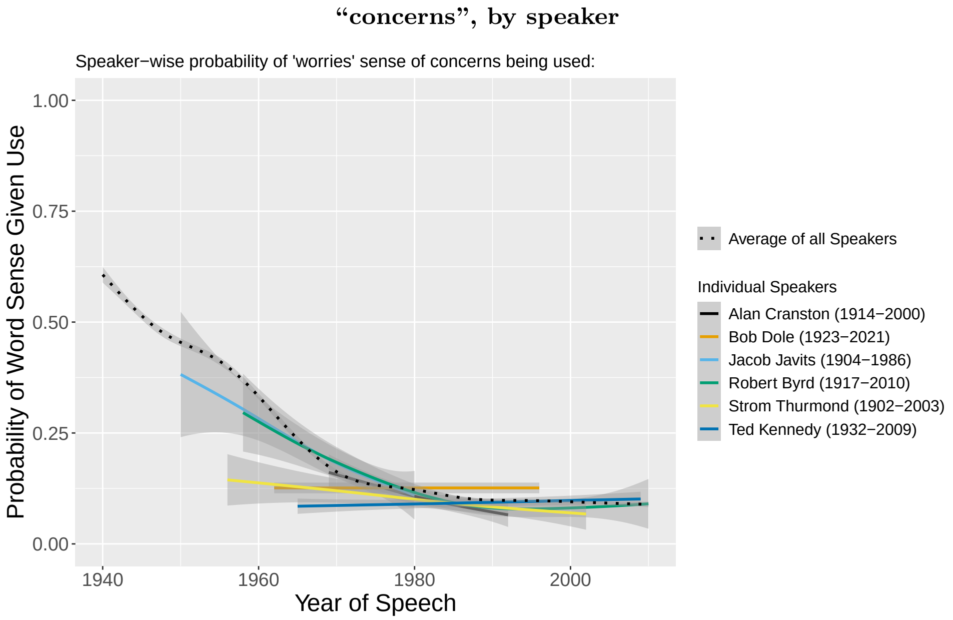

Fig. S52. "concerns"

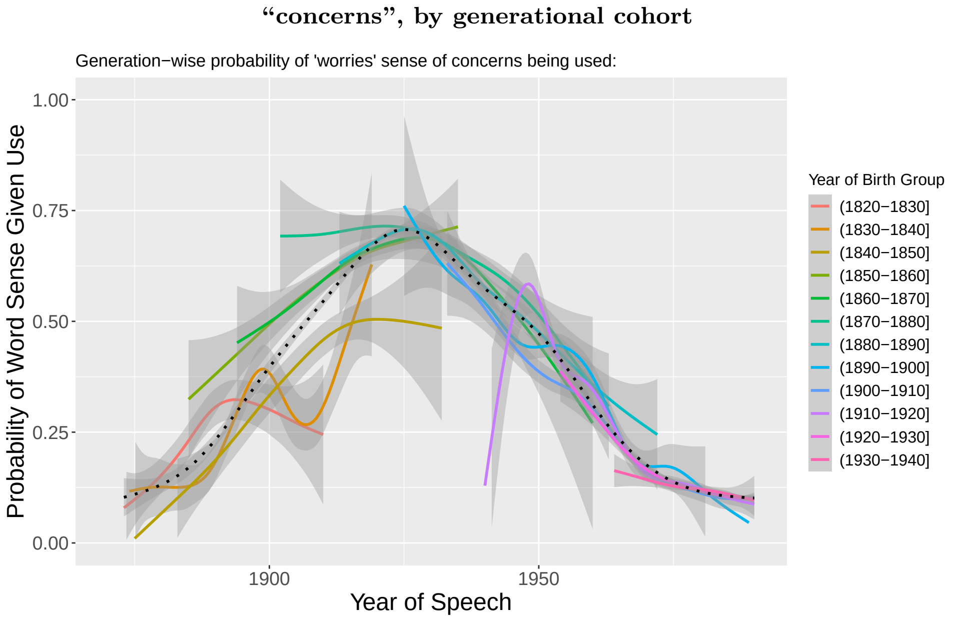

Fig. S53. "concerns"

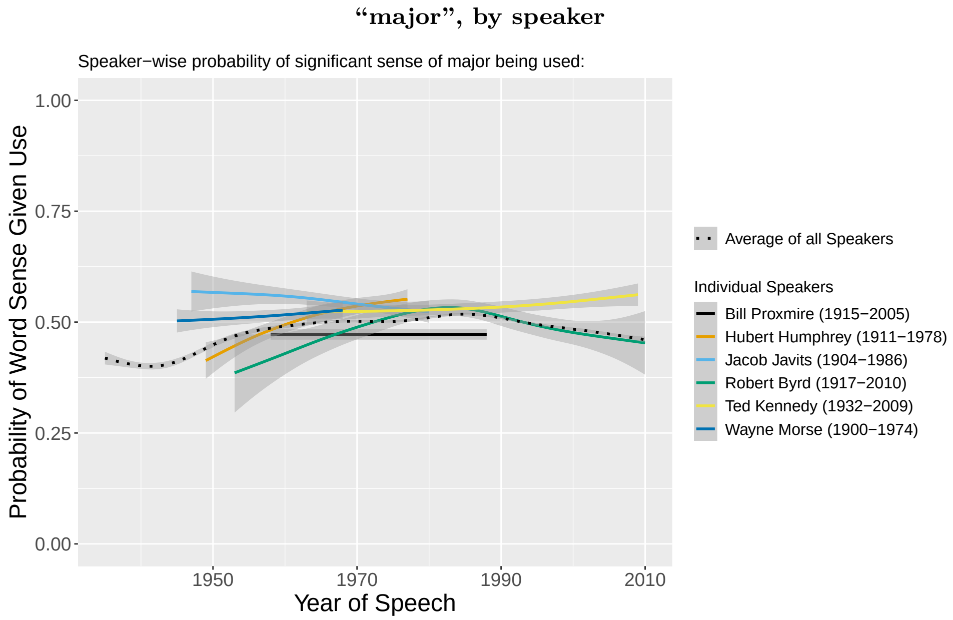

Fig. S54. “major”

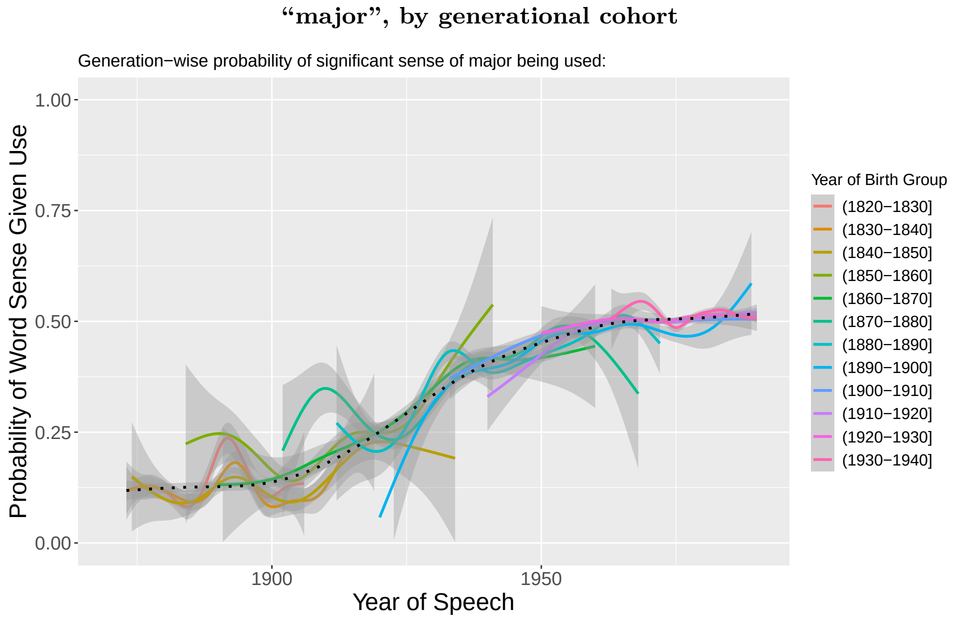

Fig. S55. “major”

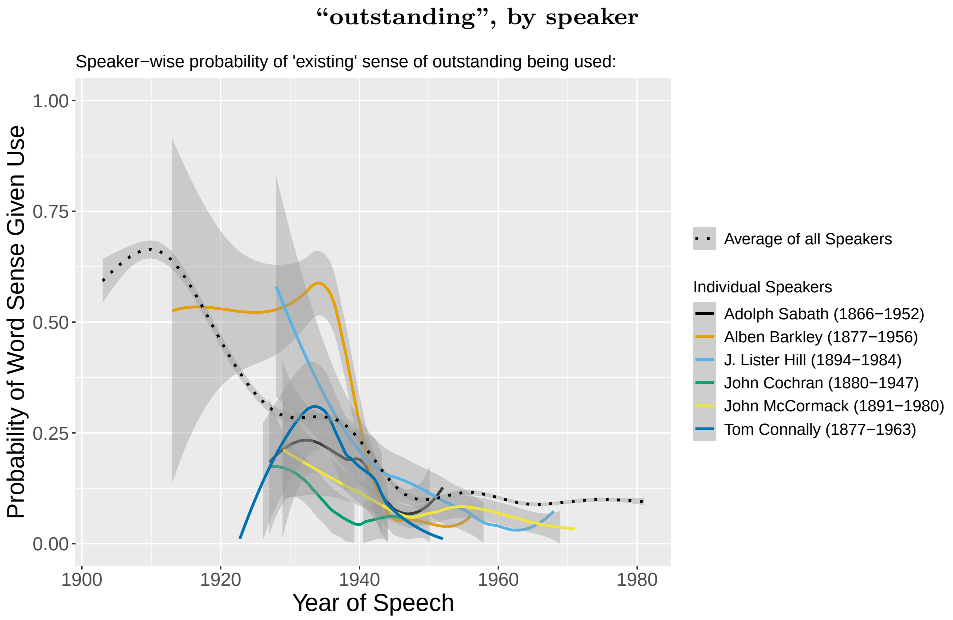

Fig. S56. “outstanding”

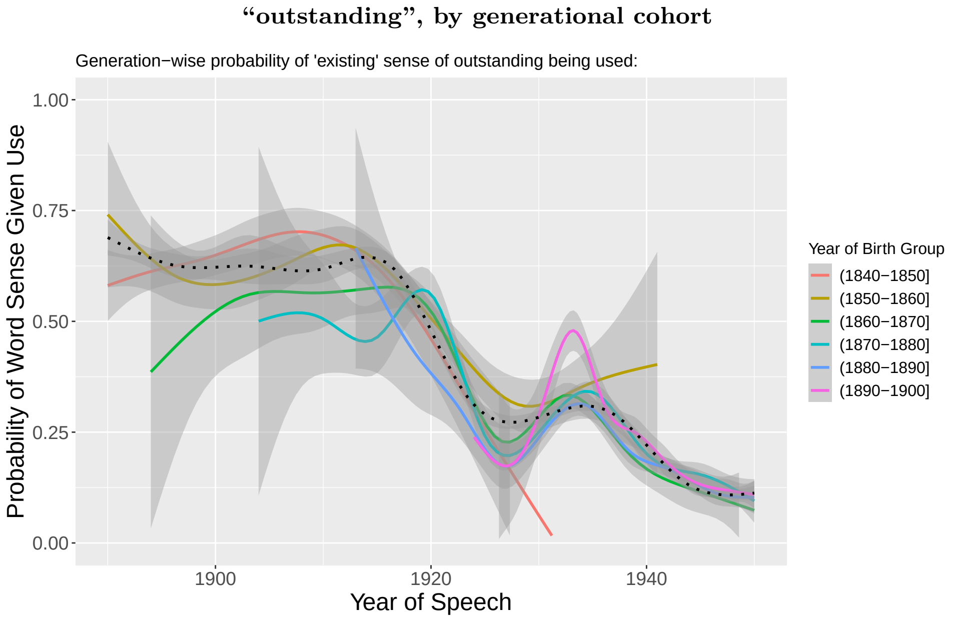

Fig. S57. “outstanding”

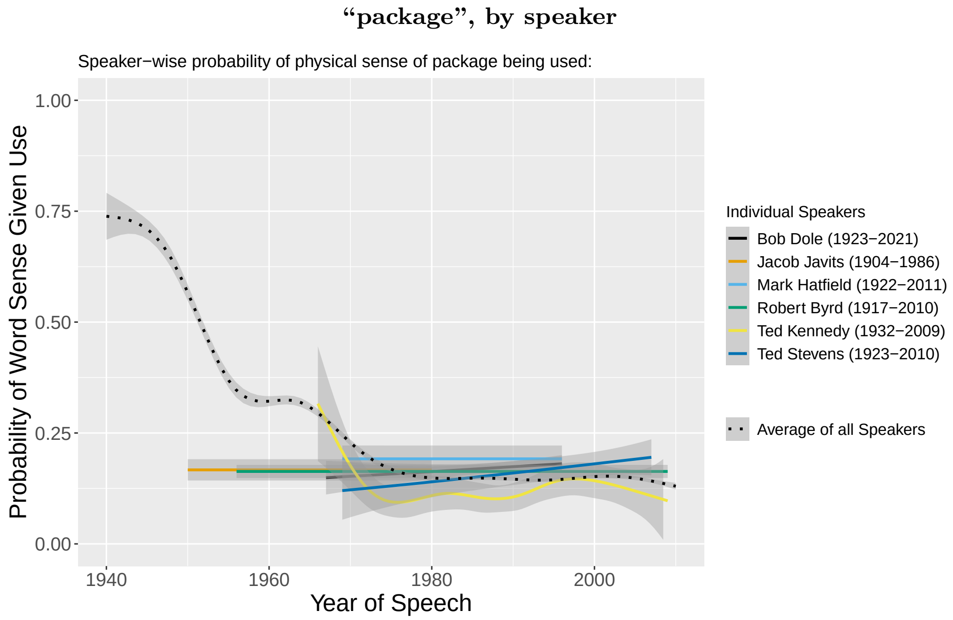

Fig. S58. “package”

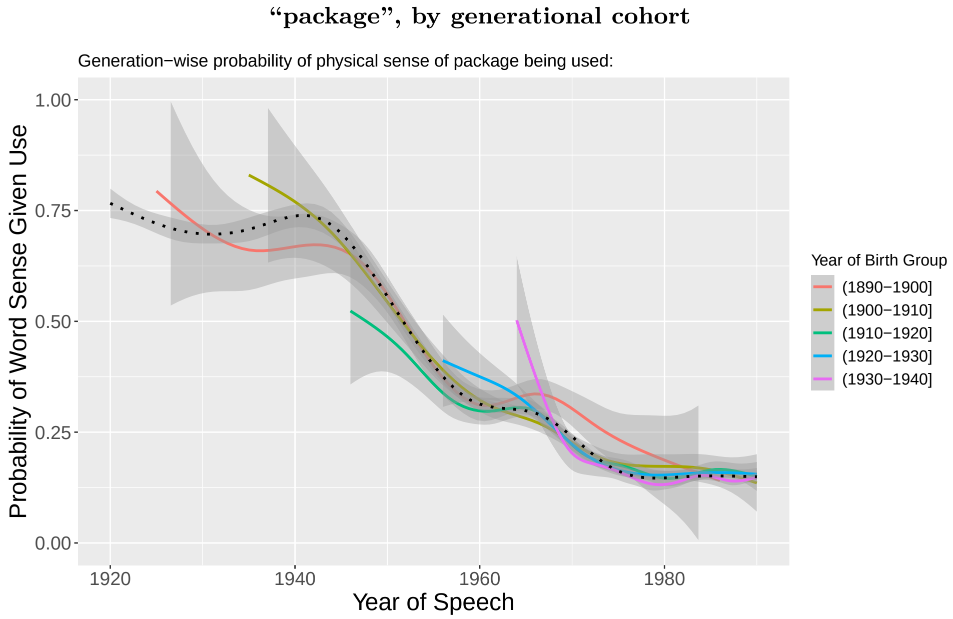

Fig. S59. “package”

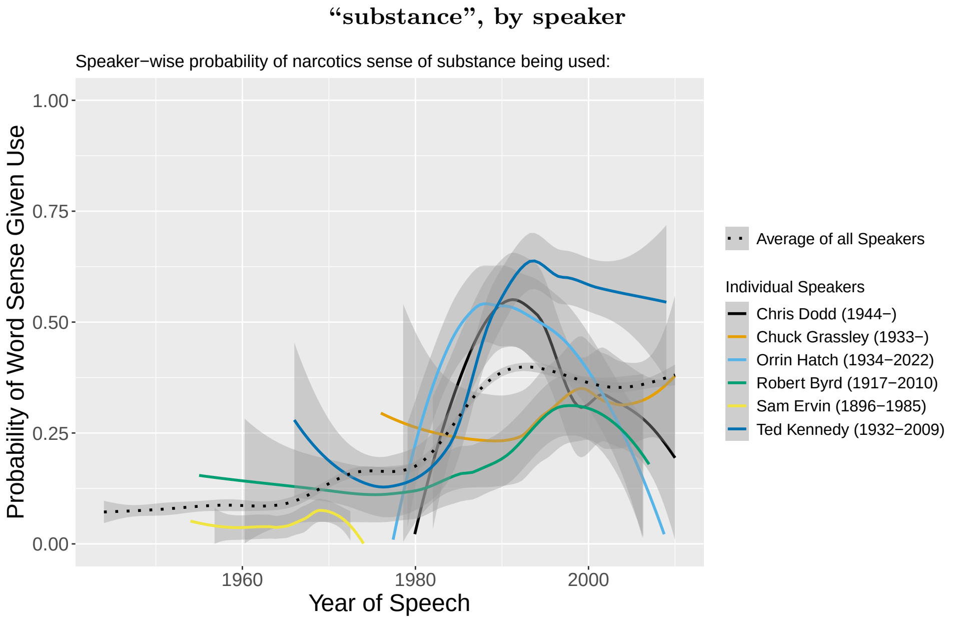

Fig. S60. "substance"

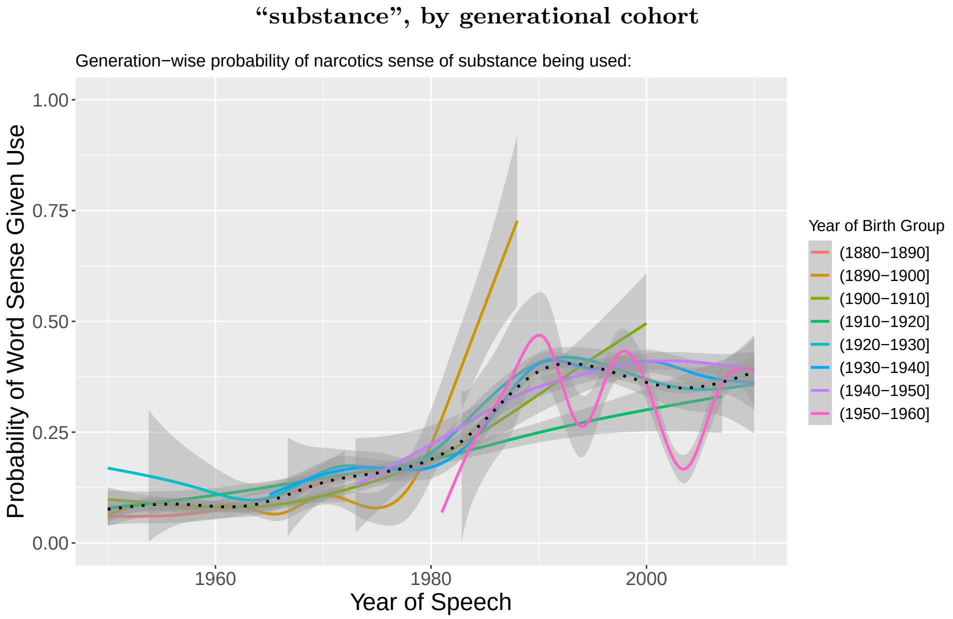

Fig. S61. "substance"

References

1. D Schlechtweg, B McGillivray, S Hengchen, H Dubossarsky, N Tahmasebi, SemEval-2020 task 1: Unsupervised lexical semantic change detection in *Proceedings of the Fourteenth Workshop on Semantic Evaluation*. pp. 1–23 (2020).
2. WL Hamilton, J Leskovec, D Jurafsky, Diachronic word embeddings reveal statistical laws of semantic change in *Proceedings of the 54th Annual Meeting of the Association for Computational Linguistics (Volume 1: Long Papers)*, eds. K Erk, NA Smith. (Association for Computational Linguistics, Berlin, Germany), pp. 1489–1501 (2016).
3. SN Wood, Fast stable restricted maximum likelihood and marginal likelihood estimation of semiparametric generalized linear models. *J. Royal Stat. Soc. (B)* **73**, 3–36 (2011).
4. R Core Team, *R: A Language and Environment for Statistical Computing* (R Foundation for Statistical Computing, Vienna, Austria), (2023).
5. PC Bürkner, Bayesian item response modeling in R with brms and Stan. *J. Stat. Softw.* **100**, 1–54 (2021).
6. B Carpenter, et al., Stan: A probabilistic programming language. *J. statistical software* **76** (2017).
